# Molecular principles underlying aggressive cancers

**DOI:** 10.1038/s41392-025-02129-7

**Published:** 2025-02-17

**Authors:** Ruth Nussinov, Bengi Ruken Yavuz, Hyunbum Jang

**Affiliations:** 1 Computational Structural Biology Section, Frederick National Laboratory for Cancer Research, Frederick, MD 21702 USA; 2 Cancer Innovation Laboratory, National Cancer Institute at Frederick, Frederick, MD 21702 USA; 3 Department of Human Molecular Genetics and Biochemistry, Sackler School of Medicine, Tel Aviv University, 69978 Tel Aviv, Israel

**Keywords:** Tumour heterogeneity, Cancer models

## Abstract

Aggressive tumors pose ultra-challenges to drug resistance. Anti-cancer treatments are often unsuccessful, and single-cell technologies to rein drug resistance mechanisms are still fruitless. The National Cancer Institute defines aggressive cancers at the tissue level, describing them as those that spread rapidly, despite severe treatment. At the molecular, foundational level, the quantitative biophysics discipline defines aggressive cancers as harboring a large number of (overexpressed, or mutated) crucial signaling proteins in major proliferation pathways populating their active conformations, primed for their signal transduction roles. This comprehensive review explores highly aggressive cancers on the foundational and cell signaling levels, focusing on the differences between highly aggressive cancers and the more treatable ones. It showcases aggressive tumors as harboring massive, cancer-promoting, catalysis-primed oncogenic proteins, especially through certain overexpression scenarios, as predisposed aggressive tumor candidates. Our examples narrate strong activation of ERK1/2, and other oncogenic proteins, through malfunctioning chromatin and crosslinked signaling, and how they activate multiple proliferation pathways. They show the increased cancer heterogeneity, plasticity, and drug resistance. Our review formulates the principles underlying cancer aggressiveness on the molecular level, discusses scenarios, and describes drug regimen (single drugs and drug combinations) for PDAC, NSCLC, CRC, HCC, breast and prostate cancers, glioblastoma, neuroblastoma, and leukemia as examples. All show overexpression scenarios of master transcription factors, transcription factors with gene fusions, copy number alterations, dysregulation of the epigenetic codes and epithelial-to-mesenchymal transitions in aggressive tumors, as well as high mutation loads of vital upstream signaling regulators, such as EGFR, c-MET, and K-Ras, befitting these principles.

## Introduction

Aggressive cancers have been associated with multiple factors.^1–5^ This comprehensive review chronicles several highly aggressive cancers, their drug regimen, and their accelerated growth and malignancy. It explores the fundamental differences between highly aggressive cancers and the less aggressive, more treatable ones. Especially, it focuses on their underlying principles on the fundamental molecular and cell signaling levels. It formulates their hallmarks and weighs the clues that they provide to anti-cancer drug combination strategies targeting their ultra-strong drug resistance. Without exceptions, the examples of aggressive cancers that it narrates—all labeled by the clinical literature as highly aggressive—reveal scenarios promoting overexpression of oncogenic proteins, suppression of tumor suppressors, and crucially, altered expression of their regulators (e.g., see refs. ^6–11^; reviewed below). Potent oncogenic mutations, especially in upstream regulators, are common. The review further discusses the outcome: a massive payload of catalysis-ready conformational states, accompanied by powerful and heterogenous cell signaling.

Tumors harboring massive increases in the numbers of active molecules of relevant oncogenic proteins, especially through certain immense overexpression frameworks, are predisposed aggressive tumor candidates. Aggressive cancers are also manipulated by strong activating mutations in proteins upstream in major proliferation pathways, such as receptor tyrosine kinases (RTKs), constitutively contributing vast numbers of active molecules.^12–15^ Spatial single-cell transcriptomics^16,17^ support the expectation that over time, over- (for suppressors, under-) expression vandalizes the signaling networks, vacating cellular controls, amplifying dedifferentiation thus heterogeneity. Mutational variants upstream follow this pattern, as observed early on in knockin *PIK3CA* mutants,^18–21^ and RTKs such as epidermal growth factor receptor (EGFR), human epidermal growth factor receptor 2 (HER2, also known as ErbB2), and mesenchymal epithelial transition factor (MET, also known as hepatocyte growth factor receptor HGFR).^22,23^ The higher the number of the oncogenic proteins, the more undifferentiated the population, and the more aggressive the tumor.^24–26^ Undifferentiated, aggressive cancer states relate to the cargo of conformationally active, oncogenic molecules.^27–33^

**Fig. 1:**
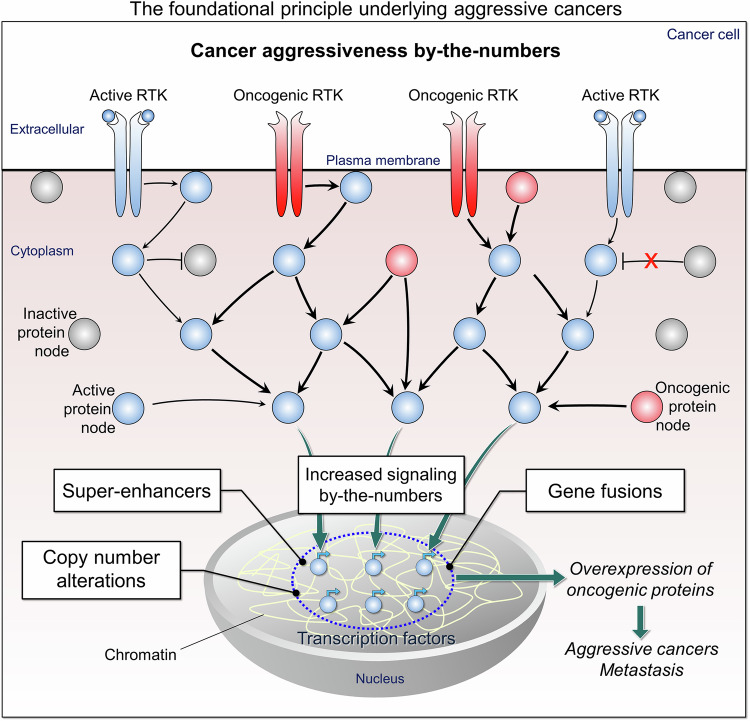
The foundational principle underlying aggressive cancers: cancer aggressiveness by-the-numbers. We propose that *the absolute number of active (oncogenic) conformations that the cancer harbors are a foundational hallmark of its aggressiveness. The higher the number—the more overspilled the signaling—the higher the heterogeneity. In aggressive cancers the number is extremely high*. *We dub this hallmark “cancer aggressiveness by-the-numbers”*. In this molecular level definition, aggressive tumor candidates are those harboring massive, catalysis-primed oncogenic proteins, produced through overexpression scenarios and strong activating driver mutations. Both generate transcriptional landscapes *signaling by-the-numbers* scenario.^14,25^ Overexpression of oncogenic proteins is caused by an increase in gene expression due to epigenetic and genetic mechanisms, involving super-enhancers, hybrid gene fusions resulting from the combination of two independent genes, copy number alterations with lost or gained DNA segments, and an increase in signaling to target genes. A high propensity of active conformations shifts the population from an inactive state (gray spheres) to a constitutively active conformation (red RTKs and spheres).^412^ This leads to an increase in the number of active molecules (blue RTKs and spheres) of the corresponding protein node, resulting in an elevation in active transcription factors (small blue spheres with arrows), which in turn leads to the overexpression of oncogenes. Our molecular level definition updates the traditional definition of the National Cancer Institute, which defines an aggressive cancer as one that “forms, grows, or spreads rapidly and requires more intensive or severe treatment than usual”. For clarity, see the section on questions and clinical implications. It defines an active protein conformation, how their number can be assessed, explains why thresholds are still unavailable and challenging to establish. It also addresses how to measure expression of super-enhancers and overexpression, and more. Tumor suppressors are not included

Escalating active oncogenic (plummeting inactive suppressor) protein loads is a foundational hallmark of aggressive cancers (Fig. 1). It is “foundational” since it is expressed on the molecular level by conformational distributions,^34–36^ the most fundamental physical-chemical attribute of biomacromolecules. Dynamic conformational, or ensemble, propensities decide molecular, and cell function.^37,38^ Factors include timing (e.g., pediatric cancers have shorter time span to evolve, thus lower acquired mutation burden,^26^ with age there are more mutations^39–42^) and the function of the mutational variants (e.g., signaling, cell cycle). They also include predisposition through *preexisting* germline mutations,^43–48^ as in familial breast, ovarian, and colorectal cancers, and preexisting or acquired glioblastoma mutations as in the apparent linkage to earlier bouts of melanomas.^49–51^ Their tissue environment (e.g., brain, pancreas), and cell states are cardinal as well.^52^

Aggressive tumors are more difficult to treat, as they are made up of multiple states and can easily transition between them. For glioblastoma, four dominant lineage specific states were defined, neural-progenitor-like, oligodendrocyte-progenitor-like, astrocyte-like, and mesenchymal-like,^53,54^ but the states are nonhomogeneous with substates between them presenting a continuum. Consistently, analysis of the spectrum of tumor aggression between cells that are likely to remain in the primary tumor and those likely to metastasize, indicated differences in gene expression patterns.^55^ Aggressive clones turned on genes associated with epithelial-to-mesenchymal transition (EMT), showing that cells exist along a continuum of EMT states. Gene signatures of late-hybrid EMT states pointed to reduced survival in both human pancreatic and lung cancer patients, highlighting their relevance to clinical disease progression. Highly aggressive tumors are characterized by large increase of catalytically primed states.^56^ In glioblastoma, driver kinases include EGFR, platelet-derived growth factor receptor α (PDGFRA), and cyclin-dependent kinase 4 (CDK4). Their mechanistic framework may offer insight into therapeutic strategies to alleviate resistance to small molecule drug combinations.^57–63^ However, it has been unclear whether the states can be separately targeted, as the transition barriers are low, thus easy to flip. The lineage specific transcription factor waves, which reprogram neuroblastoma from self-renewal to differentiation,^64^ were also offered as targetable. The low barriers argue that the multitude of cell states in primary tumors would populate metastases of highly aggressive tumors too, challenging targeting.^65–70^

The absolute number of active (oncogenic) conformations that the cancer harbors can be a foundational hallmark of its aggressiveness (Fig. 1). The higher the number—the more the oncogenic signaling activates multiple pathways—the higher the heterogeneity. We call this molecular level hallmark “cancer aggressiveness by-the-numbers”. Below, we discuss highly aggressive tumor types and scenarios with altered transcriptomic landscapes, with examples. Lower grade tumors developing into highly aggressive forms harness these scenarios. Followed over time, spatial single-cell transcriptomics^71^ can help decipher mechanisms that tumor cells adopt toward therapeutic management.

Below, we focus on neuroblastoma, glioblastoma, pancreatic ductal adenocarcinoma (PDAC), non-small cell lung cancer (NSCLC), and leukemia. In the section on clinical research progress targeting the molecular mechanisms and targets of aggressive cancers, we add to these breast, liver, and colorectal cancers (CRC).

## The dedifferentiated cancer state

Highly aggressive tumors are typically in undifferentiated or poorly differentiated states.^72,73^ They tend to grow rapidly and metastasize. The common postulate adopted by the National Cancer Institute is that the more abnormal the cells look under a microscope, the more aggressive the cancer and the faster it is likely to grow and spread. The differentiation stage of tumors is a key factor in histopathological classification of solid malignancies. A disorganized tumor under the microscope is more aggressive than a more differentiated tumor.^74^

The loss of organization and smooth borders in undifferentiated states are the outcome of corrupt protein-protein interaction networks which result from lopsided patterns of gene expression, normally regulated via homeostasis mechanisms. Grossly skewed over- (under-) expression of oncogenic genes, resulting from mutations and epigenetic alterations acquired during cancer evolution result in non-physiological patterns of genome expression, degrading the functional proteome.^75–79^ Normal developmental programs are temporally regulated by a network of interactions. The cells cascade down Waddington progenitor self-renewal growth states, exiting to accomplish complete differentiation.^80–83^ In contrast, rapidly proliferating mutant microclusters relapse into dedifferentiated populations,^84–86^ resembling earlier developmental states. In such lineage backsliding, cells do not revert to their original genome expression states. Instead, their dysregulated chromatin gains a high capacity to grow and proliferate. The cells are confined to Waddington wells, held captive by high energy mountains.

**Fig. 2:**
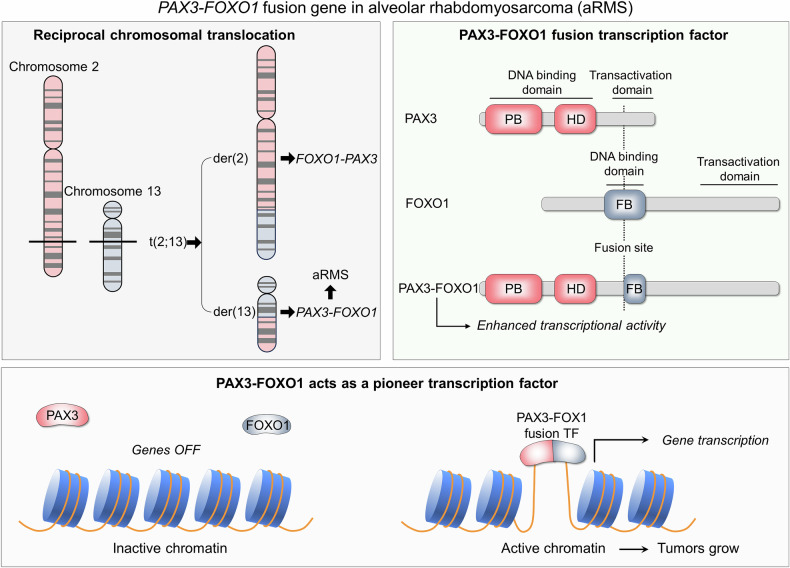
*PAX3*-*FOXO1* fusion gene in rhabdomyosarcoma (RMS). RMS rare type of cancer that can be highly aggressive. It starts as a growth of cells in the soft tissue. There are several types of RMS, including embryonal (eRMS) and alveolar (aRMS), with aRMS being the far more aggressive. The *PAX3*-*FOXO1* fusion gene is a signature genetic alteration for aRMS. It consists of a stable reciprocal translocation of chromosomes 2 and 13, t(2;13), which generates two derivative chromosomes, der(2) and der(13) (top left panel). The der(2) chromosome contains the *FOXO1*-*PAK3* fusion gene, which encodes a protein lacking major functional domains, and the der(13) chromosome contains the *PAX3*-*FOXO1* fusion gene, which encodes the PAX3-FOXO1 fusion transcription factor with enhanced transcriptional activity (top right panel). The *PAX3* gene encodes a member of the PAX family of transcription factors. FOXO1 is also a transcription factor. In the PAX3-FOXO1 fusion, the in-frame DNA binding domain of PAX3 is fused with the transactivation domain of FOXO1, generating a transcription factor with powerful transcriptional power, altered post-translational regulation, and possibly new targets.^88^ As a pioneer factor, PAX3-FOXO1 alters the local chromatin structure and binding to repressed, inaccessible chromatin, and transcriptional activation (bottom panel). PAX3 paired box 3, FOXO1 forkhead box O1

Function is the attribute of the differentiated state. Undifferentiated scenarios include amplified mutational load in key nodes, and rampant overexpression. As detailed in the examples below, certain overexpression scenarios appear predisposed to encode more aggressive tumors. Overexpression can be through super-enhancers, dysregulated epigenetic marks, especially associated with the transcription machinery, fusion of transcription factors, copy numbers of target genes, and signaling nodes, such as RTKs (Fig. 1). Aggressive, overexpressed pediatric tumors (e.g., PAX3-FOXO1 fusion-positive alveolar rhabdomyosarcoma) (Fig. 2), which are distinguished from the milder variant (embryonal rhabdomyosarcomas) provide a good example.^87,88^ Embryonal rhabdomyosarcomas do not harbor overexpression (under-expression) scenarios, nor does it display high mutation burden. Different than adult cancers, the relatively short developmental time span precludes high mutational burden populations. Instead, like other aggressive pediatric tumors, alveolar rhabdomyosarcoma harnesses an aberrant expression scenario, in this case an epigenetic mechanism, histone lysine demethylases. Epigenetically dysregulated super-enhancers in neuroblastoma and medulloblastoma provide additional examples.^64,89,90^

Aberrant gene expression biases the cellular networks.^91^ Through crosslinked pathways, the oncogenic cargo corrupts multiple proliferation pathways, escalating self-renewal of nonfunctional, undifferentiated cell states, tumor progression, and treatment failure.^54,92^ Below, we discuss metastasis and follow with overexpression scenarios in aggressive cancers. We further suggest protocols for selection of drug combinations,^57–59^ and the underlying concept.

## Aggressive cancers tend to metastasize

Apart from glioblastoma, the cancers described above undergo metastasis, a hallmark of aggressive cancer. Invasive growth sets the stage for metastatic dissemination.^93^ Invasion is a multi-step process. Cancer cells break away from a primary tumor mass and invade the surrounding stroma. Cascading metastasis events include diffusion, entering, and exiting dormancy, migration, and settling in distant organs.^94^ These capabilities can be gained by clonal selection from the heterogeneous genetic (e.g., mutational) states in the primary cancer cell population, and from transitioning states through epigenetic alterations and integration into the immune environment. Glioblastoma’s star-shaped astrocytes were offered as one reason for evading metastasis. The frequent metastasis of primary tumors to the brain, such as lung cancer, breast cancer and melanoma, were suggested to be the outcome of more productive pharmacology in those tumors as compare to glioblastoma.^95–98^ The longer elapsed time due to the effective drug regimen leads to emergence of additional resistant mutations, while the heterogeneously challenging blood-brain barrier compromises effective targeting of brain metastases. The process of metastasis raises fundamental questions, including a better grasp of the steps that are involved, their requirements, the proteins and cell states which are involved, and more, especially those related to pharmacology.^98–100^

Considering the connection between embryo development and cancer, proliferation and migration are not unique to metastasis. Embryonal brain development also involves proliferation of cells prior to acquiring fully differentiated cell states and migration. As in cancer, glial cells migrate individually or as groups,^101–103^ a process which is required for cortical development.^102^ In tumors, EMT cancer cells^104^ can spread as single cells via mesenchymal or amoeboid modes or move as groups, or microclusters.^84–86,105^ In tumors, cells clusters produce the growth factor epigen, observed in the nanolumina in cell-cell junction spaces, suggested to assist in migration, and as such, as therapeutic target.^86^ Circulating tumor cell clusters in breast cancer are up to 100 times more metastatic than single tumor cell, with the clustered cells displaying adhesion, epigenetic alterations and plasticity, pointing to high oncogenic and migration potential.^106^ Questions relating to how cells break away,^104^ how they seed secondary sites and how persist there, may relate to the embryonic brain too.

## Oncogenic expression scenarios

Cell differentiation is controlled by dynamically balanced gene regulatory networks. The networks are influenced by lineage, state specific master transcription factors,^64,107^ which decide gene expression. While lineage populations over developmental time have not been quantified,^108^ distinct cell lineages likely accommodate nonidentical distributions, separated by Waddington energy barriers.^81^ Expression levels are determined by chromatin accessibility, which is governed by epigenetics. They are influenced by super-enhancers, which are clusters of enhancers whose activity is controlled by epigenetics marks.^109^ Temporal histone decorations decompress chromatin. Organized by scaffolding proteins, which can, e.g., catalyze the epigenetic marks of mono-, di-, or tri-methylation at lysine 4 of the peptide tails of histone H3, as a core component of the Set1/mixed-lineage leukemia (MLL) histone methyltransferase complexes,^110^ such as protein lysine methyltransferase G9a,^111^ result in spatially proximal, organized, master transcription factors. These bind to the super-enhancers and to other regulatory elements, driving stronger expression of their target genes. Cell type is manipulated by the combination of the active enhancers, thus master transcription factors.^112^ Exactly how the super-enhancer circuitry transitions the cell from a self-renewal state to a differentiated state is still unclear.^64^ But the resulting unrestricted overexpression spills over to connected pathways, activating them, and pushing the cell toward the dedifferentiated state—which is the core mechanism harnessed by aggressive cancers.^113^

Highly aggressive cancers include PDAC, NSCLC, CRC, hepatocellular carcinoma (HCC) or liver cancer, breast cancer, prostate cancer, and brain tumors, such as glioblastoma. Neuroblastoma is an example of a highly aggressive solid tumor in early childhood. Below we consider their oncogenic expression scenarios, starting with neuroblastoma.

### Neuroblastoma

**Fig. 3:**
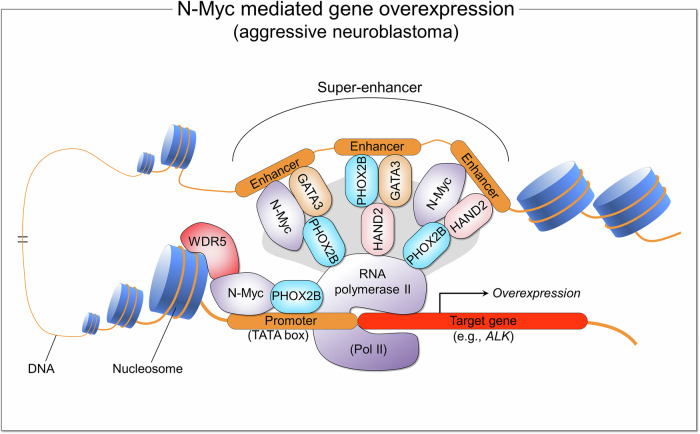
Schematic diagram of N-Myc mediated gene overexpression in aggressive neuroblastoma. Neuroblastoma is a rare pediatric cancer that develops in the nervous system of infants and children. It affects immature nerve tissue (neuroblasts) in the adrenal glands. The *MYCN* gene is amplified in multiple neuronal and nonneuronal tumors.^139^ Among these, its amplification, which encodes the N-Myc transcription factor, is a key prognostic factor in neuroblastoma. N-Myc binds to available promoters containing the TATA box with the help of WDR5, a conserved regulator of gene expression.^142^ Binding upregulates canonical Myc target genes (e.g., *ALK*), activating and promoting RNA polymerase II (Pol II), driving oncogenic gene expression and cell proliferation. Transcription factors PHOX2B, HAND2 and GATA3 support N-Myc’s binding to the super-enhancers and promoter,^131^ and subsequent gene expression. Super-enhancers are formed by multiple enhancers. The gray area represents the mediator complex, which regulates transcription by connecting enhancers to promoters

Neuroblastoma is a clinically heterogenous pediatric cancer. It can be aggressive, with diverse efforts and in-depth explorations under way.^114–120^ These include revealing its heterogeneity and clonal distributions,^121–123^ neuronal-like differentiation by downregulating CRMP5,^124^ and overexpression driving *MYC*-like gene expression.^125^ TAF1D transcriptionally activates G_2_/M phase-related genes in *MYCN* (a member of the *MYC* oncogene family encoding the transcription factor N-Myc), amplifying neuroblastoma,^126^ invasion and metastasis.^127–131^ Different from the differentiated phenotypic form,^64,132–134^ the aggressive, high-risk variant results from genetic and epigenic aberrations, driving high aberrant transcriptional output, dysregulated transcriptome, and the undifferentiated state.^135^ Neuroblastoma originates from the developing peripheral sympathetic nervous system^64,136^ and develops outside the brain and spinal cord—most commonly in or around the adrenal glands, on top of the kidneys.^64^ Embryonic neural crest cells proximal to the neural tube migrate and generate the ganglia of the peripheral sympathetic nervous system and the adrenal medulla.^137^ Genes that regulate the cell cycle (e.g., Liu et al.^138^) are overexpressed and commonly include *MYCN*,^64,139^ often with copy number alterations (CNAs). Elevated transcription promotes cell proliferation, stalling sympathoadrenal progenitor cells differentiating.^140^ N-Myc drives oncogenesis by cooperating with the G9a (also known as euchromatic histone-lysine N-methyltransferase 2, EHMT2) histone methyltransferase and the WD repeat-containing protein 5 (WDR5) adapter to mastermind global gene transcription.^141,142^ WDR5 assists N-Myc to bind promoters and up-regulate canonical Myc target genes to stimulate cell proliferation, whereas N-Myc recruits G9a to enhancers to down-regulate neuronal differentiation genes and inhibit cell differentiation.^141^ Crucial factors in aggressive neuroblastoma include core master transcription factor regulators of the neuroblastoma lineage PHOX2B, GATA3, and HAND2,^64,143–145^ with their cell type-specific overexpression determined by super-enhancers and N-Myc,^131^ whose levels in neuroblastoma migrating neural crest cells promote proliferation but restrain differentiation (Fig. 3).

Pediatric tumors are driven by relatively few genetic aberrations,^135^ carrying large genomic rearrangements and chromosomal CNAs, coupled with mutations in tumor suppressors or tumorigenic transcription factors, such as N-Myc. Considering their short evolution time, this is expected. In aggressive high-grade gliomas (pHGG), overexpression is controlled by histone methylation epigenetic alterations.^146–152^ In alveolar rhabdomyosarcoma, overexpression is via the PAX3-FOXO1 pioneer transcription factors gene fusion (Fig. 2).^88,153^ TRIB3 silencing promotes the downregulation of the AKT pathway and PAX3-FOXO1 in high-risk rhabdomyosarcoma.^154^

Among the remarkable innovative findings, is the potential of retinoic acid in neuroblastoma pharmacology.^64^ Retinoic acid, a derivative of vitamin A, can reprogram neuroblastoma cells and cause them to differentiate to neurons. This finding was recently highlighted at the National Cancer Institute.^155^ The most common combination of approved drugs against neuroblastoma includes cisplatin, carboplatin (Paraplatin), cyclophosphamide (Cytoxan), doxorubicin (Adriamycin), vincristine (Oncovin) and etoposide (Vepesid), but others may be used. For children in the high-risk group, other drugs might be added as well, and some drugs might be given at higher doses.^156^ Recently, a two hits rational dual strategy was proposed to more efficiently counter neuroblastoma,^157^ where a chimeric antigen receptor (CAR)-T cell treatment appears more efficient by increasing the cell surface expression of the CAR target structure via a small molecule.^158–160^ CAR-T cells targeting anaplastic lymphoma kinase (ALK), which is frequently highly expressed on the surface of neuroblastoma cells, eliminate the tumor cells.

Additional targeting explorations include TIM-3 blockade,^161^ a potential targetable mutation,^162^ targeting immune checkpoint,^163^ and its migration, invasion and metastasis,^164^ warning about the consequences of radiation,^165,166^ and chemotherapy, ALK-related neuroblastic tumor susceptibility, targeting c-Myc transactivation, immune modulation, prognosis, suppressor of ferroptosis, prevention, and more.^167–170^

### Glioblastoma

Glioblastoma, the deadliest brain cancer, was proposed to be spatially organized by neurodevelopmental programs, mimic glial-like wound healing, and characterized by substantial heterogeneity.^171,172^ Its dynamic organization, including proliferating and differentiated cells, retains the hierarchy of normal brain development. Glioblastoma’s tumor cells epitomize neural cell types.^54,173^ Their relative frequencies are influenced by the copy number of *CDK4*, *EGFR*, and *PDGFRA* and mutations in *NF1*. Glioblastoma aggressiveness, and therapeutic failure, are rooted in its excessive transcriptional heterogeneity, resulting from overexpression and activating mutations in the proteins it targets in the cell cycle and proliferative signaling.^54,174^ Its variability is intra-tumoral, temporal, and influenced by therapy.^175^ The states are plastic, encompassing multiple transitional microstates, and the heterogeneity is impacted the tumor’s developmental state, which embraces early embryonic neural development. Glioblastoma’s mammoth transcriptomic heterogeneity emerges via specific core genetic events that corrupt cell signaling, epigenetic, developmental, and microenvironmental origins. Within this framework, the immense amplification of the mitogen-activated protein kinase (MAPK) pathway, including via EGFR and PDGFRA, plays a cardinal role. *EGFR* gene amplifications are associated with astrocyte-like (AC-like) cells, *CDK4* amplification with neural-progenitor-like (NPC-like), and *PDGFRA* with oligodendrocyte-progenitor-like (OPC-like) states. Severe oncogenic phenotype in patients with large deletions of *NF1* Chr5q (chromosome 5q) point to higher frequency of mesenchymal-like (MES-like) states, leading to increased plasticity, pro-metastatic traits such as increased motility, invasiveness, and immune system evasion, typically observed in the pro-neural subtype. Mesenchymal transformation has been dubbed the Rosetta stone of glioblastoma pathogenesis.^176^

**Fig. 4:**
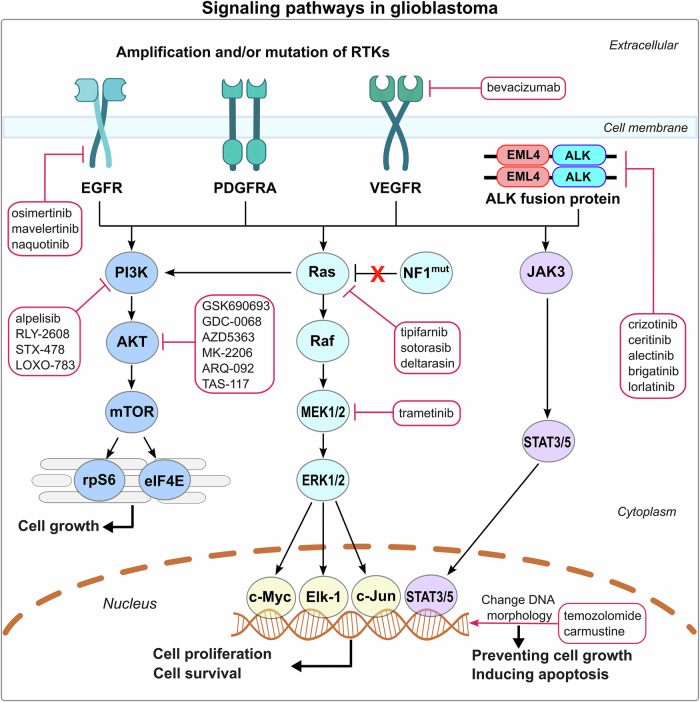
Signaling pathways in glioblastoma. PI3K/AKT, MAPK, and JAK/STAT signaling pathways orchestrate cell growth, proliferation, survival, and apoptosis. Their dysregulation is a critical driver of glioblastoma aggressiveness. In the PI3K/AKT pathway, downstream targets like ribosomal proteins rpS6 and eIF4E foster cell growth through mTOR signaling. Loss-of-function mutations in the tumor suppressor NF1 abrogate its inhibitory role on Ras, leading to overactivation of the MAPK pathway. The MAPK and JAK/STAT pathways regulate cell proliferation and survival via the transcription factors such as c-Myc, Elk-1, c-Jun, and STAT3/5. VEGFR vascular endothelial growth factor receptor, EML4 echinoderm microtubule-associated protein-like 4, eIF4E eukaryotic translation initiation factor 4E

We provide an overview of the signaling pathways whose aberration and overexpression contribute to glioblastoma aggressiveness along with some drugs targeting these pathways (Fig. 4). Overexpression resulting from strong amplification or activating mutations in RTKs, such as EGFR, PDGFRα, VEGFR, and ALK fusion protein with echinoderm microtubule-associated protein-like 4 (EML4), triggers overspilled signaling through pathway crosstalk, promoting enormous heterogeneity, driving dedifferentiation and drug resistance. Several therapeutic agents have been identified that target key components of these signaling pathways. For instance, EGFR is targeted by small molecule inhibitors such as osimertinib (Tagrisso), mavelertinib (PF-06747775), and naquotinib (ASP8273), while VEGFR is targeted by the monoclonal antibody bevacizumab (Avastin, Mvasi, Zirabev). ALK fusion proteins can be inhibited by agents like crizotinib (Xalkori), and ceritinib (Zykadia), alectinib (Alecensa), brigatinib (Alunbrig), and lorlatinib (Lorbrena). PI3K inhibitors include alpelisib (Piqray), RLY-2608, STX-478, and LOXO-783, while AKT inhibitors encompass GSK690693, GDC-0068, AZD5363, MK-2206, ARQ-092, and TAS-117. Additionally, Ras inhibitors such as tipifarnib (Zarnestra), sotorasib (Lumakras), and deltarasin, alongside the downstream MEK inhibitor trametinib (Mekinist), serve to attenuate oncogenic signaling. For chemotherapeutic agents specific to brain cancers, temozolomide (Temodar) prevents cancer cells from making DNA, leading to cell cycle arrest at G2/M and apoptosis, and carmustine (BiCNU, Gliadel) cross-links DNA and RNA, resulting in inhibition of DNA synthesis, RNA production and RNA translation.

By corrupting cell signaling, overexpression of these RTKs alters the landscape of the cellular states, enforcing tumor development. Overexpression of EGFR in nestin^+^ neural progenitors drive astrocytoma-like tumors; PDGF/PDGFR manipulate oligodendroglioma-like tumors,^54,177^ activating primarily the Janus kinase/signal transducer and activator of transcription 3 (JAK/STAT3), phosphoinositide 3-kinase/protein kinase B/mammalian target of rapamycin (PI3K/AKT/mTOR), Ras/MAPK, and transforming growth factor β (TGFβ) pathways (Fig. 4). In glioblastoma, EPHA2, an RTK, was observed to mediate PDGFA/PDGFRA signaling, and EGFR, a major driver of MAPK, activates glutamate dehydrogenase (GDH1) transcription to promote glutamine metabolism through the mitogen-activated protein kinase kinase/extracellular signal-regulated kinase/ETS domain-containing protein Elk-1 (MEK/ERK/Elk-1) pathway.^178^ The outcome is biased signaling, skewed toward different dedifferentiated cell states, whose relative frequencies reflect the amplification genetic events. The multiple affected tumor pathways scenarios argue why combination therapy targeting multiple pathways is essential.

Currently, there is no small drug treatment approach that will be effective for every glioblastoma. Vorasidenib had positive results in delaying progression of a specific form of glioma.^179,180^ It doubled progression-free survival in people with recurrent grade 2 glioma with isocitrate dehydrogenase 1 (IDH1) or 2 (IDH2) mutations. Not a small molecule, but important to cite, is intrathecal bivalent CAR-T cells targeting EGFR and interleukin-13 receptor α2 (IL-13Rα2), which is undergoing clinical trials in recurrent glioblastoma.^181^ It is a dual-target treatment, with CAR-T cells targeting proteins common in brain tumors: EGFR (in 60% of the patients), and IL-13Rα2 (in 75%).^182^ A tested drug combination includes indotecan (LMP400, a topoisomerase I inhibitor) and niraparib (PARP inhibitor, prevents DNA repair, leading to cell death).^183^ Drugs used to treat glioblastoma multiforme^184^ include temozolomide (Temodar), bevacizumab (Avastin, Mvasi, Zirabev), procarbazine hydrochloride (Matulane), hydroxyurea (Hydroxycarbamide, Droxia, Siklos), carmustine (BiCNU, Gliadel), bevacizumab-maly (Alymsys), bevacizumab-tnjn (Avzivi), and bevacizumab-adcd (Vegzelma).

Additional glioblastoma mechanistic studies were carried out.^185,186^ Pharmacological explorations include degradation^185^ and WDR1-dependent cytoskeleton remodeling.^187^ They target its signaling,^188–190^ starve it, target its polymerase, and its epigenetic STING modulation.^191^ They also undertake prognosis,^192^ target the immune checkpoint,^193,194^ immunotherapy,^195,196^ its invasion via EMT, take up peptide-based inhibition strategy,^197^ microRNAs, chemotherapy, and more.^198–200^ Glioblastoma heterogeneity has been undertaken through network modeling and a systems-level approaches, which identify shared and tumor-specific signaling alterations, such as MEK1 activation, NUMB variability, and 3D mutation patches, ultimately stratifying patients into groups with distinct survival outcomes and advancing network-guided precision medicine.^201,202^ Several single-cell and spatial analysis studies have been aiming to map and analyze the spatial heterogeneity of glioblastoma to reveal tumor evolution, regional molecular specificity, and potential therapeutic targets.^172,203–205^

### Pancreatic ductal adenocarcinoma (PDAC)

**Fig. 5:**
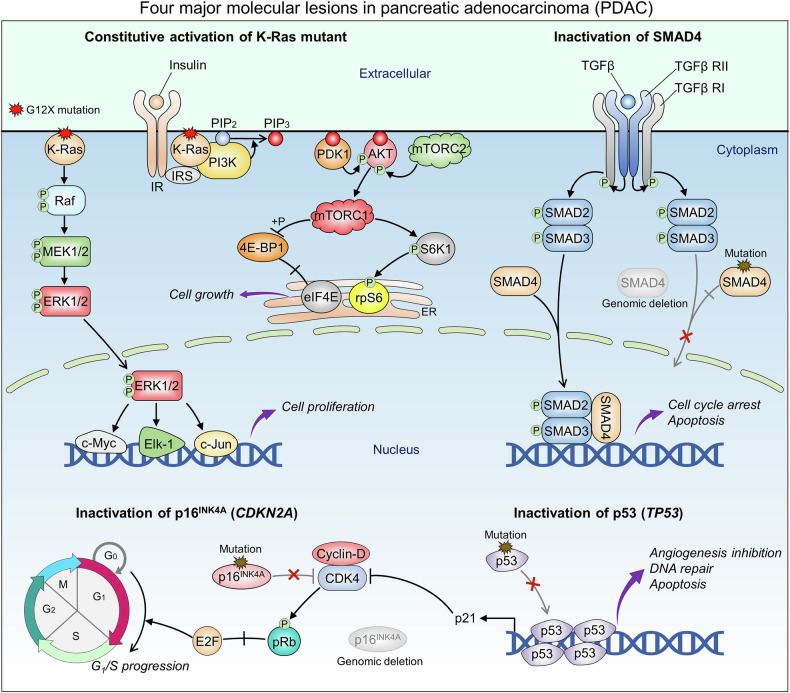
Schematic representation of four major molecular lesions in pancreatic ductal adenocarcinoma (PDAC). PDAC is primarily caused by genetic mutations in four genes: an oncogene, *KRAS* (encoding K-Ras); and three tumor suppressor genes, *TP53* (encoding p53, a transcription factor), *CDKN2A* (encoding p16^INK4A^, a CDK inhibitor), and *SMAD4* (encoding SMAD4, a transcription factor). Constitutively active K-Ras with the G12X mutation leads to increased activation of Raf, MEK, and ERK through a phosphorylation cascade. ERK activates transcription factors such as c-Myc, Elk-1, and c-Jun, leading to cell proliferation. Insulin binding to IR together with active K-Ras initiates PI3K activation. PI3K converts PIP_2_ to PIP_3_, leading to mTORC1 activation. This includes AKT activation by PDK1 and mTORC2. mTORC1 phosphorylates S6K1 and 4E-BP1. S6K1 activates rpS6. Phosphorylation of 4E-BP1 removes its inhibitory role on eIF4E, which is involved in translational activation and regulation of cell growth. Inactivation of p16^INK4A^ by mutation or genomic deletion impairs its function as a CDK4 inhibitor, leading to an unregulated cell cycle transition. Inactivation of p53 by mutation hinders its functions, such as blocking of angiogenesis, DNA repair, and induction of apoptosis. p53 mutant also impairs the expression of p21 (a CDK inhibitor), which is involved in G_1_/S arrest due to damaged DNA. The TGFβ ligand binds to the TGFβ receptor type II (RII) dimer, which recruit the type I (RI) dimer to form a hetero-tetrameric complex. RII phosphorylates the serine/threonine kinase of RI. Under physiological conditions, RI phosphorylates the receptor-regulated SMAD (RSMAD), such as SMAD2 and SMAD3, causing them to dissociate from the receptor complex. The RSMAD complex associates with a common mediator SMAD (coSMAD), i.e., SMAD4, to form a complex that enters the nucleus to bind its target genes, leading to cell cycle arrest and apoptosis. Inactivation of SMAD4 by mutation or genomic deletion impairs its tumor suppressor function in PADC. As discussed in the text, as a highly aggressive cancer, it also involves overexpression of multiple genes, e.g., *YAP1*, *MYC*, *HMGA2*, *IGF2BP1* and *IGF2BP3*, and dysregulation of epigenetics modulators, e.g., HDAC1/2/5, KDM6A, MLL histone methylases, and histone methyltransferases. IR insulin receptor, PDK1 phosphoinositide-dependent kinase 1, 4E-BP1 eukaryotic translation initiation factor 4E (eIF4E)-binding protein 1, IRS insulin receptor substrate

While the nature of PDAC cell of origin is unclear,^206^ early emergence of mutations in *KRAS* [G12D (39%), G12V (32%), G12R (17%)] in plastic pancreatic exocrine cells is a hallmark of PDAC.^207^ The mutations occur in early stages,^208^ initiating and maintaining PDAC development. High frequencies of strong *KRAS* mutations testify to potent, heterogenous signaling output through multiple effector pathways (Fig. 5). Further contributing to basal cell transitions and intrapopulation heterogeneity are the mutations in *CDKN2A* (a cyclin-dependent kinase inhibitor), *TP53* (a tumor suppressor), and *SMAD4* (a transcription factor mediating TGFβ signaling), all associated with intraepithelial neoplasia (PanIN), the dominant precursor of PDAC.^209^ Overexpression of the yes-associated protein 1 (YAP1) transcription factor^210^ and the frequently mutated epigenetic regulatory genes, including histone modification enzymes, overexpression of the histone deacetylase 5 (HDAC5),^211^ aberrations in MLL histone methylases, histone methyltransferases, and the lysine demethylase 6A (KDM6A) histone demethylase, a potent tumor repressor,^212–215^ which is associated with histone 3 lysine 4 (H3K4) methylation and histone 3 lysine 27 (H3K27) demethylation,^216^ aggressively increase the transitions and heterogeneity. Tumors with genetic defects in MLLs are likely to induce expression of chromatin-regulating genes and cell proliferation-associated genes (including SWI/SNF chromatin remodeling complexes, and their components) and genes involved in cell cycle progression and proliferation.^215^ HDAC1 and HDAC2 are also highly expressed in pancreatic cancer, and can be recruited to the epithelial-cadherin (CDH1) promoter (involved in histone deacetylation) by zinc-finger E-box binding homeobox 1 (ZEB1), thereby promoting EMT and tumor metastasis.^217,218^ SIRT6 (Sirtuin 6) histone deacetylase ablation can promote PDAC metastasis by hyperacetylating histone 3 lysine 9 (H3K9) and histone 3 lysine 56 (H3K56), leading to Myc recruitment. These events result in overexpression of high-mobility group AT-hook 2 (HMGA2), insulin-like growth factor 2 mRNA-binding proteins (IGF2BP1 and IGF2BP3), downstream of let-7.^219^ Overexpression of c-Myc is crucial hallmark of aggressive cancer cells.^220,221^ Recently it was also observed K-Ras-GTP inhibition in pancreatic cancer.^222^ The overexpression patterns of Myc, IGF2BP2, a tumor promoter that drives cancer proliferation through its client mRNAs IGF2 and HMGA1, which regulates the expression of replication-dependent histone genes and the cell-cycle in cancer cells, and epigenetics gene aberrations, couple with overactive signaling pathways in pancreatic cancer tumorigenesis and metastasis, including the MAPK, PI3K/AKT, NF-κB, JAK/STAT, Hippo/YAP, and Wnt pathways. Further, YAP, a transcriptional coactivator along with transcriptional co-activator with PDZ binding motif (TAZ) and transcriptional enhanced associate domain (TEAD) is also overexpressed.^211,222–224^

Through gains in copy numbers, overexpressed transcription factors and their activators upregulate transcription of multiple genes, thereby promoting EMT. Key signaling nodes like AKT have been pointed as harboring aberrant expression, as are epigenetics-related proteins, such as HDAC1 and HDAC2, MLL histone methylases, histone methyltransferases, and the KDM6A histone demethylase, and more. These combine with *RAS, CDKN2A*, *TP53*, and *SMAD4* high oncogenic mutational burden (Fig. 5), collectively resulting in multiple distorted signaling pathways, pushing tumor cells dedifferentiation. Altogether, these stand out, clarifying the aggressive behavior of PDAC, which can be attributed to the multiple powerful overexpression scenarios, promoting EMT, and shifts in cell states.

Drug resistance to RMC-7977, is a highly selective inhibitor of active K-Ras, H-Ras, and N-Ras, resulted in detected Myc copy number gain, which could be overcome by combinatorial TEAD inhibition in vitro.^222^ c-Myc overexpression is common in PDAC. It binds to promoters, interacts with proliferative pathways in pancreatic cancer, boosts aerobic glycolysis and regulates glutamate biosynthesis, thus cancer cells metabolism, contributing to EMT, and aggressive PDAC behavior.^220^ Small drug therapies approved for pancreatic cancer^225^ include paclitaxel (Abraxane, Taxol), everolimus (Afinitor, Votubia, Zortress), capecitabine (Xeloda), erlotinib hydrochloride (Tarceva), 5-fluorouracil or 5-FU (Carac, Tolak, Efudex, Fluoroplex), gemcitabine hydrochloride (Gemzar, Infugem), irinotecan hydrochloride (Campto, Camptosar, Onivyde), olaparib (Lynparza), mitomycin (Mitosol, Mutamycin), and sunitinib malate (Sutent).

Additional exploratory therapeutic approaches target reprogramming of cancer-associated fibroblasts, heterogeneity, metastatic pancreatic cancer,^226^ degradation,^227^ neddylation,^228^ nutrient restriction, vascular endothelial growth factor (VEGF)-independent angiogenesis, targeting ferroptosis, signaling,^229^ metabolic dependencies,^230^ chemotherapy-induced anti-tumor immunity to boost anti-PD-1 therapy, modulating the immune response including immune checkpoint therapy, prognosis, EMT-associated chemoresistance, prevention, enhancing proteasome inhibition, IL-33, and more, altogether, broad translational challenges and trends.

### Non-small cell lung cancer (NSCLC)

Lung cancer is a leading cause of cancer-related mortality.^231^ NSCLC is more common, especially lung adenocarcinoma (LUAD), and less aggressive than small cell lung cancer (SCLC). Both metastasize quickly, often to the brain.^232–235^ Its expression patterns were comprehensively analyzed,^236^ and its major signaling tracked to RTK, MAPK, PI3K/AKT/mTOR, JAK/STAT, apoptosis [B cell lymphoma protein, Bcl-2-associated X protein, first apoptosis signal ligand (FasL)], Notch, Hedgehog, Wnt, and the YAP/TAZ/TEAD Hippo pathways.^237,238^

**Fig. 6:**
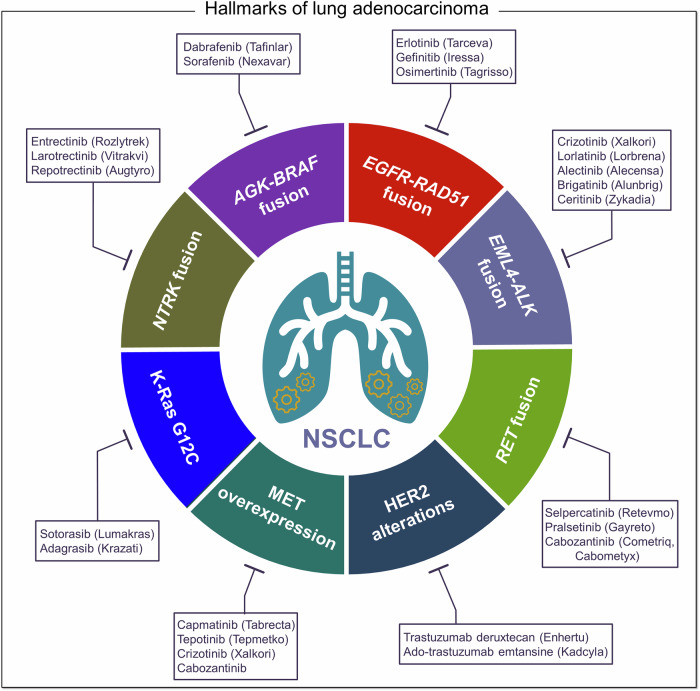
Hallmarks of lung adenocarcinoma. Overexpression of active conformations such as catalytically primed kinases is associated with non-small cell lung cancer (NSCLC). These increased kinase activities are attributed to fusion genes, including *NTRK* fusion, *AGK*-*BRAF* fusion, *EGFR*-*RAD51* fusion, *EML4*-*ALK* fusion, and RET fusion genes (see text for details). NSCLC also harbors HER2 alterations, MET overexpression, and K-Ras G12C mutation. Generic names of small drugs that inhibit these active molecules are shown in the figure with the brand name in parentheses

NSCLC tumors commonly harbor overexpression of diverse catalytically primed kinases (Fig. 6). Scenarios include *NTRK* fusion genes, such as *NTRK1*, *NTRK2*, and *NTRK3* (encoding tropomyosin receptor kinases, TrkA, TrkB, and TrkC, respectively),^239,240^ that link the tyrosine kinase domain with a 5’ fusion partner (over 50 partners across cancers). With no ligand binding domain, the resulting constitutively activated Trk promotes tumor cell proliferation. A second scenario includes 5´ *BRAF* fusion partners, the most frequent being *AGK* (a gene encoding acylglycerol kinase) in LUAD.^241–243^ Most are in-frame with the B-Raf kinase domain,^242,243^ with the N-terminal Ras binding domain (an autoinhibitory domain) truncated,^244^ making B-Raf and the MAPK pathway constitutively active. In a third scenario, NSCLC harnesses metastatic EGFR fusions,^245^ where the tyrosine kinase domain is frequently fused to RAD51, a protein involved in DNA damage response. The missing EGFR autophosphorylation and adapter binding sites at its C-terminal tail are replaced by EGFR’s Tyr845 in the EGFR kinase domain-RAD51 fused protein.^245,246^ The loss of tyrosine 1045, which acts in EGFR degradation, extends the protein half-life time. Additional kinase fusion scenarios include the LUAD ALK-positive fusion often with EML4.^247,248^ HER2 tyrosine kinase receptor also undergoes alterations, including amplification, mutations, and overexpression.^249^ Rearranged during transfection (RET) overexpression is frequent in lung neuroendocrine tumors and is associated with response to RET tyrosine kinase inhibitors.^250^ MET overexpression ranges in from 15 to 70% of NSCLC patients.^251^ MET oncogenic mechanisms include fusions, mutations in the tyrosine kinase domain, and exon 14 skipping alterations.^252^ c-MET is an RTK for hepatocyte growth factor (HGF). Like some other RTKs, c-MET activates multiple pathways, including MAPK and PI3K/AKT/mTOR. Activating mutations, such as MET exon 14 skipping mutations (METex14), cause hyper-activation, thus proliferation, EMT, and metastasis, especially to the brain,^232,253^ previously associated with a poor prognosis, currently targeted with capmatinib (Tabrecta).^254^

All RTKs sustain residue mutations (recently reviewed by Waarts et al.^255^). In drug resistance the alterations can collaborate, as observed in the METex14 driver mutation co-existing with amplification of CDK4 (which inactivates retinoblastoma, a regulator of cell proliferation) and MDM2 (inactivates p53), PDGFR, and FGFR. Other possible alterations include EGFR, PDGFR, RET, and K-Ras. In individual patients a large majority of driver gene mutations are homogeneous across all metastases,^256^ but rare subclonal alterations are heterogeneous in different metastasis.^232,257^ These suggest that a single anti-resistance drug cannot slow tumor growth. However, combined with drugs targeting the dominant driver mutations (e.g., MET14ex), may extend progression-free and overall survival.^232^ Amplification of Myc and TERT (telomerase reverse transcriptase) appears common events in lung cancer patients. This is not surprising since Myc copy number change also emerges in drug resistance to K-Ras mutations, as shown recently in targeting K-Ras^G12C^ in pancreatic cancers.^258^

Small drug therapy for NSCLC includes cisplatin, carboplatin (Paraplatin), pemetrexed (Alimta), paclitaxel (Abraxane, Taxol), docetaxel (Docefrez, Taxotere), gemcitabine hydrochloride (Gemzar, Infugem), and vinorelbine (Navelbine).^259^ Angiogenesis inhibitors including bevacizumab (Avastin, Mvasi, Zirabev) are used in combination with chemotherapy, immunotherapy, or erlotinib (Tarceva) in advanced or metastatic NSCLC. Ramucirumab (Cyramza) is used in combination with the targeted drug erlotinib (Tarceva) or chemotherapy advanced or metastatic NSCLC. Specific targeting of oncogenic proteins includes K-Ras inhibitors, sotorasib (Lumakras) for advanced NSCLC with the K-Ras G12C mutation.^260^ Adagrasib (Krazati) resembles sotorasib. EGFR inhibitors targeting cells with either an exon 19 or exon 21 mutation include osimertinib (Tagrisso), afatinib (Gilotrif), erlotinib (Tarceva), dacomitinib (Vizimpro), gefitinib (Iressa), or erlotinib (Tarceva) in combination with a VEGF inhibitor. EGFR inhibitors that target cells with S768I, L861Q and/or G719X mutations include afatinib (Gilotrif) or osimertinib (Tagrisso), and erlotinib (Tarceva), dacomitinib (Vizimpro), and gefitinib (Iressa). EGFR inhibitors that target cells with an exon 20 mutation include amivantamab (Rybrevant) monoclonal antibody. ALK inhibitors include lorlatinib (Lorbrena) and second-generation ALK inhibitors alectinib (Alecensa), brigatinib (Alunbrig), and ceritinib (Zykadia). ROS1 inhibitors include entrectinib (Rozlytrek), crizotinib (Xalkori), and ceritinib (Zykadia). A B-Raf inhibitor, dabrafenib (Tafinlar), is used in combination with trametinib (Mekinist), a MEK inhibitor, and encorafenib (Braftovi) with binimetinib (Mektovi), also a MEK inhibitor. Vemurafenib (Zelboraf) and dabrafenib (Tafinlar) can be single treatment medications. RET inhibitors including selpercatinib (Retevmo) and pralsetinib (Gayreto), and cabozantinib (Cometriq, Cabometyx) act against RET, ROS1, MET, and VEGF. MET inhibitors including capmatinib (Tabrecta), tepotinib (Tepmetko), and crizotinib (Xalkori) act against MET, ALK, and ROS1. HER2-directed drugs include fam-trastuzumab deruxtecan-nxki (Enhertu) and ado-trastuzumab emtansine (Kadcyla). The examples above include sotorasib (Lumakras) for NSCLC K-Ras G12C mutation,^260^ which can couple with upstream SHP2 inhibitor vociprotafib (RMC-4630). Notably, sotorasib plus panitumumab (Vectibix) are used in refractory colorectal cancer.^261^

Experimental approaches explore heterogeneity,^262^ EMT,^263^ antibody-drug conjugate in advanced HER2-mutant NSCLC, combinations,^264,265^ immunotherapy modulation, including immune checkpoint,^265^ RTK resistance, mutations, adverse effects, signaling,^266^ metastasis, genome instability, prognosis, apoptosis, degradation, and more, providing an overview. Recently, a combined inhibition of K-Ras^G12C^ and mTORC1 kinase was also offered.^266^

Additional solid tumors that can be aggressive include HCC or liver cancer, colorectal cancer, breast cancer, and prostate cancer.

### Leukemia

Acute leukemia (AL) can be an example of a blood cancer^267^ and includes acute lymphoblastic leukemia (ALL) and acute myeloid leukemia (AML). Unlike chronic lymphocytic leukemia (CLL) and chronic myelogenous leukemia (CML), which progress slowly, both ALL and AML progress rapidly. AL results from clonal proliferation of myeloid and lymphoid progenitor cells.^268^ Acute promyelocytic leukemia (APL, the most curable AML subtype) has been labeled a paradigm for targeted differentiation therapy.^269^ APL results from a t(15;17)(q22;q21) translocation that fuses *RARA* (a gene encoding retinoic acid receptor α, RARα) on 17q21 to *PML* (a gene encoding promyelocytic leukemia protein, PML) on 15q22. It generates two fusion genes,^270^ which express PML–RARα and a reciprocal RARα–PML, an aberrant retinoid receptor. PML–RARα heterodimerizes with the retinoid X receptor (RXR), binding to retinoic acid-responsive elements, stalling myeloid differentiation. The resulting high population of promyelocytes expresses and binds factor VII (proconvertin), activating factor X (Stuart-Prower factor) and factor IX (antihemophilic factor B), and pro-coagulant states.^271,272^

AML is the most aggressive adult leukemia, with clonal differentiation arrest of progenitor or precursor hematopoietic cells.^273^ It initiates in the myeloid cells of the bone marrow and spreads into the blood. Overexpression of homeobox protein Hox-A9 (HOXA9), a pioneer transcription factor in myeloid and B progenitor cells, is key to its aggressiveness.^274^ Under physiological conditions, the activity of HOXA9 is primarily regulated by histone methyltransferase MLL3/MLL4. HOXA9-binding sites are enriched with H3K4me1 and H3K27ac, with low levels of H3K27me3. Increased H3K27me3 and H3K4me3 depress binding.^275^ Deletion of MLL3/MLL4 methylases prevents H3K4 methylation and HOXA9-promoted leukemia.^274^ The primary H3K27me3 “writer” protein, enhancer of zeste homolog 2 (EZH2), is a component of the polycomb repressor complex 2 (PRC2). Mutations in epigenetic factors are frequent in hematological malignancies.^276,277^ Lower expression of EZH2 and chromosomal translocations leading to MLL-fusion proteins activate HOXA9 expression through dysregulated chromatin modification.^278^ Fusion with nuclear pore complex protein NUP98 and overexpression of *CDX2* and *CDX4* further upregulate HOXA9.^276^ Thus, AML’s overexpressed transcription factors, and its altered epigenetic landscape and associated transcriptomic program not only harness an embryonic transcriptional development program. It also clarifies the distinction between the milder APL and the aggressive AML.

An excellent overview of targeting mutations in cancer includes a useful detailed compilation of genetic indications for targeted therapy in cancer and the timeline of their approval by the FDA.^255^ We refer the readers to this review and to the websites listed here for more detailed information. On a different note, it was observed that common anti-cancer therapies induce somatic mutations in stem cells of healthy tissue.^279^ As the authors note, being toxic to cells, chemotherapies can also increase the mutational burden of long-lived normal stem cells increasing the risk for developing second cancers.

Experimental exploratory approaches include PI3K inhibition,^280^ signaling,^280,281^ transcription factors, programmed cell death,^282^ tyrosine kinase,^283^ immune modulation,^284^ prognosis, and more.^285^

## Learning from aggressive cancers

Above, we discussed examples of highly aggressive, often deadly cancers. Our goals were two-fold: First, since they are at the tip of cancer aggressiveness, we deemed that they could inform key properties that are responsible for cancers’ capabilities, enabling forecasting cancers evolution. Second, more importantly, this knowledge may suggest a more informed pharmacological regimen, especially involving treatment combinations. Cancer is a complex disease driven by numerous factors, including environment, lifestyle, and genetic make-up.^286–289^ Some cancers are established to be rooted in familial predispositions, such as breast^290–294^ and colon^295,296^ cancers. Others are not. Yet, this premise may change with future discoveries. Cancer is not a single-mutation event.^26,297–302^ Multiple mutations are involved, with the minimal number still being debated. Rare, unidentified familial mutations can be expected to preexist, and to collaborate with emerging mutations during life, contributing to cancers, which to date are not thought to be familial. Such scenarios were also proposed to be the case in neurodevelopmental disorders and pathologies.^302–308^

*Here we ask what we can learn from highly aggressive cancers*. Especially, *can they inform the foundational hallmark*—*on the molecular level*—*underlying them*? In 2000, Hanahan and Weinberg articulated the hallmarks of cancer.^309^ A decade later they expanded the list.^310^ The impact on cancer biology was massive, as can be assessed by the tens of thousands works that related to them (e.g., see refs. ^310–316^). Hanahan and Weinberg created a monumental, visionary and practical, framework that organizes the distinctive properties evolved by cancer cells, thereby conceptualizing cancer biology.^309^ The six hallmarks of cancer that they laid out in 2020 included evading apoptosis, self-sufficiency in growth signals, insensitivity to antigrowth signals, sustained angiogenesis, tissue invasion and metastasis, and limitless replicative potential. These capture perpetuating proliferative signaling through enhanced activity of the signal transduction pathways by mechanisms including mutations, amplifications, evading growth suppressors, resisting cell death, enabling replicative immortality, inducing angiogenesis, and activating invasion and metastasis.^309^ In 2011, they added cancer abetting genome instability and mutations and tumor-promoting inflammation as enabling characteristics, and deregulating cellular energetics and avoiding immune destruction as emerging hallmarks.^310^ These capture multiple hallmark functions, alongside reprogramming energy metabolism and evading immune destruction.

The hallmarks underscore the significance of cancer rewiring normal developmental programs, adapting and sabotaging embryonic regulated cell proliferation, migration, polarity, apoptosis, and differentiation,^317–321^ and deceiving homeostasis.^13,322^ Cancer cells acquire the potential to proliferate and survive, with rare mutations and epigenetic alterations selected in drug resistance.^323–329^ Subsequently, they were further discussed by Hanahan,^330^ who explained his perspective, that in conceptualizing the hallmarks, they produced a heuristic tool that can clarify the complexity of cancer—its phenotypes and genotypes.^331^ That is, the set of hallmarks produced the simplest, and most important, facts and theories relating to cancer, which are not obvious, but detectable. He further elaborated on cancer’s phenotypic plasticity, the disrupted capability of differentiation, and the significance of epigenetic reprogramming and the microbiomes, both not included earlier, but established in the literature,^332–334^ as well as senescence and the tumor microenvironment.^93,335–339^

A modified hallmarks model suggested that invasion and metastasis should be the center of attention,^340^ making it a potentially highly ranked therapeutic target. The model argued that therapies aiming at other hallmark-specific mechanisms affect cell viability directly, however, the cancer cells population still undergoes selection and Darwinian evolution. On the other hand, obstructing metastasis stalls seeding—thus distant colonies, which is responsible for most cancer mortalities.

In line with this view, discussions of the hallmarks^341^ highlighted sustained proliferative signaling. It was argued that with cancer cells stimulating their own growth, they no longer depend on external signals—but largely on two major proliferative pathways: PI3K/AKT/mTOR and MAPK/ERK. Both consist of kinase cascades, and under physiological conditions, both are activated by stimulated RTKs. PI3K, AKT, and mTOR kinases are crucial in metabolism, growth, proliferation, survival, transcription, and protein synthesis.^342–348^ These kinases are also major regulators of cell survival by blocking apoptosis. The MAPK pathway is the major signaling cascade in cell proliferation.^15,349–353^ It transmits and augments survival signals from the cell surface to the nucleus. It is also vital to cell growth. Its stimulation activates ERK, p38 MAPK, and JNK, resulting in activation of transcription factors, including c-Jun, Myc, Elk, ETS, and ATF, and in turn, cell growth, survival, repair, and proliferation. Temporal and spatial topography of cell proliferation in cancer observed that proliferative architecture is organized at two spatial scales: large domains, and smaller niches enriched for specific immune lineages,^354^ capturing clinically significant features of cancer proliferation.^351,355–359^

## Molecular principles underlying aggressive cancers

*What is then the foundational principle*—*on the molecular, conformational level*—*underlying aggressive cancers*? Going back to the aggressive cancers’ examples discussed above, all harness mechanisms unleashing immense amplification of destructive signaling. Their actions are amplified through topographies such as their organizations in super-enhancers, fusions, alteration of copy numbers and making their enhancer and promoters accessible through epigenetics modulations (Fig. 1). Some transcription factors whose contributions are massive are dubbed master factors, others, pioneers. We also see extensive mutations in multiple relevant RTKs, and their amplifications. Mutant, dysregulated RTKs pump down high, incessant signaling volumes. Other key upstream nodes, like Ras, are mutated as well. These commonly co-occur multiple times as described in the examples. Taken together, *aggressive cancers are characterized by heavy loads of active* proteins, especially kinases, key signaling nodes, and missing, or inactive tumor suppressors. Under physiological conditions, mitogen-promoted signaling strength and duration can control cell cycle decisions; to proliferate or differentiate.^12^ Strong and short encode proliferation, weaker and sustained differentiation. In cancer, the scenarios above lead to hyper-strong, long-lived proliferation. *Signaling has been described phenomenologically, as propagating from one node to another*.^14,360–365^ Its impact on metabolism and as therapeutic targets has been reviewed, and signaling-based combinatorial treatments were offered.^57–59,366^ How cancer cells can send wrong signals was also deliberated, examples of signaling in cancer were described,^367–371^ and an overview of signaling pathways in cancer was provided. This overview highlighted the consequences of overexpressed signaling components causing dysregulation of cellular signaling, through cross talk.^372,373^ At the fundamental level, *we formulated a signaling by-the-numbers* model, which also considers the *cell type* (a skin cell differs from a brain cell) and *timing* (here, of cancer evolution). Such model considers the total, absolute numbers of active conformations of the mutant protein, the types, and locations of all its co-existing mutations, and the expression levels of specific isoforms of genes and regulators of proteins in the pathway. We suggest that tumors harboring massive, cancer-promoting, *catalysis-primed oncogenic payloads of active protein conformations, especially through certain overexpression scenarios, are predisposed aggressive tumor candidates*. The stronger the combination of driver mutations, and the bigger the number of the respective proteins, and of those who transmit the signal down the pathway, the stronger the signal transduction.

*This leads us to designate superstrong proliferative signaling as* the *molecular principle underlying aggressive cancers*, *making it a foundational hallmark*. Superstrong signaling boosts invasion and metastasis, making it a major cancer capability, providing the basis on the molecular level of these fundamental cancer phenotypes.^340^ Tissue invasion and metastasis, and limitless replicative potential capabilities require formidable activity of the signal transduction pathways.

As to pharmacology, it substantiates the call to invest in invasion and metastasis. Glioblastoma is an invasive brain tumor. Other highly aggressive cancers metastasize. Yet, to date few treatments were developed,^374^ including few suggested combinations.^375–379^ Often, metastases drugs in the chemotherapy cocktail are the same as those against the corresponding primary tumors, rather than metastases, as in metastatic breast cancer. Drugging proteins associated with metastasis is challenging. One example relates to late-stage metastatic melanoma, recently suggested to be hallmarked by low expression of postsynaptic cell adhesion molecule neuroligin 4X (NLGN4X), a suppressor, leading to HIF1A accumulation, and acquisition of migratory properties.^380^

## Questions and clinical implications of the model

Here we suggested a model of “cancer aggressiveness by-the-numbers.” Questions and implications for the clinics include:

1. *Is there a numerical threshold for high aggressiveness?* Determining a threshold is a vastly important aim—but to date, a still unattained challenge. Having such a number could help diagnose emerging cancer mutations and proliferation *prior to* observable phenotypic change.
2. *Would the threshold be the same for different types of cancers?* As to threshold consistency across cancer types, since the number depends on the strength of the mutations, extent of overexpression (e.g., gene duplication, super-enhancers, combination of genetic and epigenetic events), and the identity of the targeted genes, which vary across cancer types and evolve with cancer stages, we expect that thresholds will vary for different cancer types.
3. *What qualifies as an active (oncogenic) conformation?* An active protein conformation is the shape that the protein takes when it is biologically active. The process of changing shape is a conformational change.
4. *How to count the active conformations?* The answer is two-fold. Both ways provide estimates. The first way is measuring indirectly, by protein activity, similar to activation of proteins by driver mutations. Driver mutations activate oncogenic proteins. They lead to a conformational change from the inactive to the active conformation. Stronger mutations are likely to have lower kinetic barriers in the transitions and more stable conformations. They are expected to have a higher fraction in the active state, making the mutations stronger. Experiments may identify a stronger signaling. The second way is direct measurement, by e.g., mapping the protein conformational ensemble by a spectroscopic method, like NMR spectroscopy, or by long timescale molecular dynamic simulations.
5. *Are all active conformations treated equally when counted?* All active conformations are treated equally. Chemistry tells us: for a protein there is a single active state, where all catalytic groups are precisely coordinated (the right angle and the right distance) for the catalytic reaction. There can however be substate variations, reducing catalytic efficiency.
6. *How are super-enhancers quantified?* The expression of super-enhancers can be measured by e.g., chromatin immunoprecipitation and sequencing (ChIP-Seq).
7. *How is protein overexpression assessed?* Protein overexpression can be assessed using a variety of methods, for example, Western blotting, In-cell western assays, and comparing normal levels with those in the mutant cell.
8. *Are tumor suppressors included in this count, and if so, how?* No, they are not included. Repressors work by tamping down on expression, thus active conformations.
9. *Is there verification and examples for the model?* Verification comes from analysis of known mechanisms of aggressive cancers. This indicates that they acquire activating mutations, overexpression, gene fusion, gene duplication, epigenetics, and more. All lead to proliferation, the outcome of strong signaling through a higher number of active proteins, e.g., in the MAPK and PI3K/AKT/mTOR pathways. Inhibitors depress catalytic activity. As to examples, one protein that we worked on recently is Bcr-Abl in chronic myeloid leukemia (CML).^381,382^ C-Abl is a protein kinase in the cytoplasm. The kinase domain is in the C-terminal region. It is allosterically regulated by myristoyl (a lipidic post-translational modification), which is covalently linked at its N-terminal tail. Bcr is a transmembrane protein on the surface of B cells. It is attached to the membrane through its N-terminal domains. Under normal conditions, Abl is autoinhibited by the myristoyl which docks into its pockets at the C-lobe kinase domain, retaining Abl in an inactive conformation. In leukemia, Abl’s C-terminal domain is fused with Bcr’s N-terminal domain, removing its capacity to be autoinhibited and making it membrane associated, in a constitutively active state. Another type of example relates to the relative concentrations of the active conformations and clinical diagnosis and treatment.^302^ In this example, SHP2 clinical phenotype—cancer or RASopathies—can be predicted by mutant conformational propensities. SHP2 phosphatase promotes full activation of the RTK-dependent Ras/MAPK pathway. Its mutations can drive cancer and RASopathies, a group of neurodevelopmental disorders. We asked how same residue mutations in SHP2 can lead to both cancer and RASopathies phenotypes, and whether we can predict what the clinical outcome will be. We observed that SHP2 clinical phenotype can be predicted by mutant conformational propensities. High propensity of the active conformation was associated with cancer, lower propensity with RASopathy, offering structural guidelines for identifying and correlating mutations with clinical outcomes, and a drug strategy.
10. *What are the limitations of this proposed model?* Changes in the number of active conformations can also take place under physiological conditions. Among these, cell type-specific expression of the protein, and of other proteins in the respective pathway, timing of activation (during embryonic development or sporadic emergence), are pivotal. Further, activation is the result of allosteric events. There can be multiple such events unrelated and related to cancer, for example, effects of the cellular environment, metabolites, and the dependence on the combination of driver mutations, in the respective proteins, and in those which transmit the signal down the pathway.
11. *How this model can be used in clinical practice?* Usefulness in clinical practice is the goal. An excessively high number of active conformations lead to more active proteins. Higher activity is associated with oncogenic events in cancer evolution. Clinically, this suggests that signaling measurements can be useful in early detection of cancer in tissue samples (biopsies), in addition to visual microscopic examination, which is not quantitative.

## Clinical targeting aggressive cancers

### Drugs and drug combinations targeting aggressive cancers

There has been clinical progress in targeting the molecular mechanisms and therapeutic targets of aggressive cancers, including clinical trials resulting in FDA-approved drugs. In addition to neuroblastoma, glioblastoma, PDAC, NSCLC, and leukemia, which we discussed above, additional aggressive cancer types, such as breast cancer, liver cancer and CRC have also been actively challenged and are described in this section. To cite examples, in early-stage triple-negative breast cancer, immunotherapy before surgery was determined as able to improve the prognosis, becoming standard-of-care. For extensive NSCLCs, the FDA approved a combination of immunotherapy and chemotherapy. These and other clinical advances have been included in the report of the American Society of Clinical Oncology.

FDA-approved drugs for neuroblastoma^383^ include cyclophosphamide, dinutuximab, doxorubicin hydrochloride, eflornithine hydrochloride (or DFMO, difluoromethylornithine), naxitamab-gqgk, and vincristine sulfate (Table 1). Dinutuximab is the first therapy specifically approved for pediatric high-risk neuroblastoma in combination with interleukin-2 (IL-2 or aldesleukin), granulocyte-macrophage colony-stimulating factor (GM-CSF), and isotretinoin.^384,385^ Naxitamab-gqgk was approved for the treatment of neuroblastoma with high refractory or relapse risk in bone marrow and/or bone in combination with GM-CSF.^386^ eflornithine was approved for reducing the risk of relapse in pediatric patients with high-risk neuroblastoma who are in remission and have completed multi-agent, multi-modality therapy.^387,388^ Drug combinations used in neuroblastoma include BuMel (busulfan + melphalan hydrochloride) and CEM (carboplatin + etoposide phosphate + melphalan hydrochloride).^383^

**Table 1:** FDA-approved drugs for neuroblastoma

Drug name (brand name)	Drug type (PubChem CID)	Cancer treatment	Mechanism of action	Route of administration
Cyclophosphamide (Cytoxan, Procytox)	Small molecule (2907)	Neuroblastoma, ALL, AMoL, AML, CLL, CML, breast cancer, HL, MM, MF, neuroblastoma, NHL, ovarian cancer, retinoblastoma	Precursor of alkylating agents of metabolite PM, leading to DNA cross-linking and cell apoptosis	Oral or intravenous
Dinutuximab (Unituxin)	Monoclonal antibody	Neuroblastoma	Binds to GD2 on the surface of cancer cells, triggering an immune response	Intravenous
Doxorubicin hydrochloride (Adriamycin, Doxil, Caelyx, Rubex, Myocet)	Small molecule (443939)	Neuroblastoma, ALL, AML, breast cancer, gastric cancer, HL, NHL, NSCLC, soft tissue and bone sarcomas, thyroid cancer, transitional cell bladder cancer, Wilms tumor	Interacting with DNA interferes with Topo II activity and prevents relegation of Topo-mediated DNA breaks, leading to inhibition of replication and transcription and induction of apoptosis.	Intravenous
Eflornithine hydrochloride or DFMO (Iwilfin, Vaniqa)	Small molecule (57004)	Neuroblastoma	Inhibits ODC, an enzyme that synthesizes a polyamine required for tumor formation and growth	Intravenous
Naxitamab-gqgk (Danyelza)	Monoclonal antibody	Neuroblastoma	Binds to GD2 on the surface of cancer cells, triggering an immune response	Intravenous
Vincristine sulfate (Oncovin, Vincasar, Marqibo, Vincrex)	Small molecule (249332)	Neuroblastoma, AL, HL, NHL, RMS, Wilms tumor	Inhibits microtubule formation in the mitotic spindle, resulting in cell division arrest at the metaphase stage	Intravenous

*AMoL* acute monocytic leukemia, *DFMO* difluoromethylornithine, *GD2* glycolipid GD2, *HL* Hodgkin lymphoma, *MF* mycosis fungoides, *MM* multiple myeloma, *NHL* non-Hodgkin lymphoma, *ODC* ornithine decarboxylase, *PM* phosphoramide mustard, *RMS* rhabdomyosarcoma, *Topo II* topoisomerase II

For glioblastoma patients, FDA-approved treatments of high-grade malignant gliomas^389^ include bevacizumab, carmustine, lomustine, temozolomide, and vorasidenib (Table 2). A form of carmustine contained in a wafer, carmustine implant (Gliadel), was also approved for the treatment of glioblastoma multiforme and is applied directly during surgery.^390^ Vorasidenib was recently approved, the first approval in decades, for patients with grade 2 gliomas harboring IDH1 or IDH2 mutations.^391^ Drug combinations used in certain types of brain tumors include PCV (procarbazine hydrochloride + lomustine + vincristine sulfate).^389^

**Table 2:** FDA-approved drugs for glioblastoma

Drug name (brand name)	Drug type (PubChem CID)	Cancer treatment	Mechanism of action	Route of administration
Bevacizumab (Avastin, Mvasi, Zirabev, Alymsys, Avzivi, Vegzelma)	Monoclonal antibody	Glioblastoma, cervical cancer, CRC, HCC, non-squamous NSCLC, RCC, EOC, FTC, PCC	Binds to VEGFR, resulting in prevention of new blood vessel formation, reduction of tumor vasculature, and reduction of tumor blood supply	Intravenous
Carmustine (BiCNU)	Small molecule (2578)	Glioblastoma and other brain tumors, HL, MM, NHL	Cross-linking of DNA and RNA, resulting in inhibition of DNA synthesis, RNA production and RNA translation	Intravenous
Lomustine (CeeNU, Gleostine)	Small molecule (3950)	Glioblastoma and other brain tumors, HL	A reactive metabolite alkylating agent causes alkylation and cross-linking of DNA and RNA	Oral
Temozolomide (Temodar)	Small molecule (5394)	Glioblastoma multiforme, anaplastic astrocytoma	Prevents cancer cells from making DNA, leading to cell cycle arrest at G2/M and apoptosis	Oral or intravenous
Vorasidenib (Voranigo)	Small molecule (117817422)	Astrocytoma, oligodendroglioma	Inhibits IDH1 and IDH2 mutants, resulting in reduced production of the oncometabolite D2HG and partial restoration of cell differentiation	Oral

*D2HG* D-2-hydroxyglutarate, *EOC* epithelial ovarian cancer, *FTC* fallopian tube cancer, *HCC* hepatocellular carcinoma, *HL* Hodgkin lymphoma, *MM* multiple myeloma, *NHL* non-Hodgkin lymphoma, *PCC* primary peritoneal cancer, *RCC* renal cell carcinoma, *VEGFR* vascular endothelial growth factor receptor

The list of drugs approved for pancreatic cancer patients^225^ includes multiple drugs (Table 3). These drugs approved for pancreatic cancer are often also administered against other cancers, such as breast and lung cancer. In addition to pancreatic cancer, paclitaxel, everolimus, capecitabine, 5-fluorouracil, gemcitabine hydrochloride, and olaparib are also approved for breast cancer. Similarly, paclitaxel, everolimus, erlotinib hydrochloride, and gemcitabine hydrochloride are also approved for NSCLC. Drugs approved for the treatment of tumors in the digestive system include everolimus, capecitabine, 5-fluorouracil, mitomycin, and sunitinib malate. Drugs approved for the treatment of other cancers include everolimus and sunitinib malate for kidney cancer; everolimus for brain cancer; capecitabine and 5-fluorouracil for CRC; gemcitabine hydrochloride and olaparib for ovarian cancer; olaparib for prostate cancer; and mitomycin for urothelial cancer. Drug combinations used in pancreatic cancer include FOLFIRINOX (leucovorin calcium + fluorouracil + irinotecan hydrochloride + oxaliplatin), GEMCITABINE-CISPLATIN (gemcitabine hydrochloride + cisplatin), GEMCITABINE-OXALIPLATIN (gemcitabine hydrochloride + oxaliplatin), and OFF (oxaliplatin + fluorouracil + leucovorin calcium (folinic acid)).^225^

**Table 3:** FDA-approved drugs for pancreatic cancer

Drug name (brand name)	Drug type (PubChem CID)	Cancer treatment	Mechanism of action	Route of administration
5-fluorouracil or 5-FU (Carac, Tolak, Efudex, Fluoroplex)	Small molecule (3385)	Pancreatic cancer, breast cancer, CRC, gastric cancer	Inhibits TS enzyme, resulting in prevention of cancer cell DNA synthesis and repair, leading to cell death	Intravenous
Capecitabine (Xeloda)	Small molecule (60953)	Pancreatic cancer, breast cancer, CRC, gastric cancer	Metabolized 5-FU inhibits DNA synthesis and repair, leading to the death of rapidly dividing cancer cells	Oral
Erlotinib hydrochloride (Tarceva)	Small molecule (176871)	Pancreatic cancer, NSCLC	Inhibits EGFR kinase	Oral
Everolimus (Afinitor, Votubia, Zortress)	Small molecule (6442177)	Pancreatic cancer, breast cancer, NSCLC, gastrointestinal cancer, RCC, SEGA	Inhibits mTOR kinase, inducing cell growth arrest and apoptosis	Oral
Gemcitabine hydrochloride (Gemzar, Infugem)	Small molecule (60749)	Pancreatic cancer, breast cancer, NSCLC, ovarian cancer	Mimics DNA and RNA building blocks and interferes with DNA synthesis to slow or stop cancer cell growth	Intravenous
Irinotecan hydrochloride (Campto, Camptosar, Onivyde)	Small molecule (74990)	Pancreatic cancer	Inhibits Topo I enzyme, resulting in replication arrest and lethal double-strand breaks in DNA, leading to apoptosis	Intravenous
Mitomycin (Mitosol, Mutamycin)	Small molecule (5746)	Pancreatic cancer, gastric cancer, urothelial cancer	A bifunctional and trifunctional alkylating agent binds to DNA, leading to cross-linking and inhibition of DNA synthesis and function	Intravenous
Olaparib (Lynparza)	Small molecule (23725625)	Pancreatic cancer, breast cancer, prostate cancer, EOC, FTC, PCC	Inhibits PARP, leading to accumulation of unrepaired single-stranded DNA breaks and formation of toxic forms of double-stranded DNA breaks	Oral
Paclitaxel albumin (Abraxane, Taxol)	Small molecule (36314)	Pancreatic cancer, breast cancer, NSCLC	Binds to the β-subunit of tubulin, promoting microtubule polymerization and stabilization, resulting in mitotic arrest	Intravenous
Sunitinib malate (Sutent)	Small molecule (6456015)	Pancreatic cancer, GIST, RCC	A kinase inhibitor of multiple RTKs, including VEGFR, PDGFR, KIT, FLT3, CSF-1R, and RET, resulting in inhibition of tumor growth, pathological angiogenesis and metastatic progression	Oral

*CSF-1R* colony-stimulating factor 1 receptor, *EOC* epithelial ovarian cancer, *FLT3* fms-like tyrosine kinase 3, *FTC* fallopian tube cancer, *GIST* gastrointestinal stromal tumor, *PARP* poly(ADP-ribose) polymerase, *PCC* primary peritoneal cancer, *RCC* renal cell carcinoma, *SEGA* subependymal giant cell astrocytoma, *Topo I* topoisomerase I, *TS* thymidylate synthase, *VEGFR* vascular endothelial growth factor receptor

For NSCLC, the FDA has approved a large number of small molecule and monoclonal antibody drugs (Table 4).^392^ These small molecule drugs target K-Ras^G12C^ (adagrasib, sotorasib); the kinase domain of multiple RTKs, including the EGFR family, MET, ALK, ROS1, IGF1R, FLT3, RET, the Trk family and the EML4-ALK fusion protein (afatinib dimaleate, alectinib, brigatinib, capmatinib hydrochloride, ceritinib, crizotinib, dacomitinib, entrectinib, erlotinib hydrochloride, gefitinib, lazertinib mesylate hydrate, lorlatinib, osimertinib mesylate, pralsetinib, repotrectinib, selpercatinib, tepotinib hydrochloride); B-Raf mutants (dabrafenib mesylate, encorafenib); MEK1/2 (binimetinib, trametinib dimethyl sulfoxide); mTOR (everolimus); tubulin (docetaxel, paclitaxel albumin, vinorelbine tartrate); enzymes responsible for nucleotide synthesis (methotrexate sodium, pemetrexed disodium); and DNA (doxorubicin hydrochloride, gemcitabine hydrochloride). The monoclonal antibodies target the extracellular domain of RTKs on the surface of tumor cells, including EGFR, HER2 and VEGFR (amivantamab-vmjw, bevacizumab, necitumumab, ramucirumab, trastuzumab) and block the receptors, programmed cell death 1 (PD-1) and cytotoxic T-lymphocyte antigen 4 (CTLA-4) on the surface of T cells (cemiplimab-rwlc, ipilimumab, nivolumab, pembrolizumab, tremelimumab-actl) and the ligand PD-L1 on the surface of tumor cells (atezolizumab, durvalumab). Drug combinations used in NSCLC include CARBOPLATIN-TAXOL (carboplatin + paclitaxel) and GEMCITABINE-CISPLATIN (gemcitabine hydrochloride + cisplatin).^392^

**Table 4:** FDA-approved drugs for NSCLC

Drug name (brand name)	Drug type (PubChem CID)	Cancer treatment	Mechanism of action	Route of administration
Adagrasib (Krazati)	Small molecule (138611145)	NSCLC, CRC	A covalent inhibitor of K-Ras^G12C^	Oral
Afatinib dimaleate (Gilotrif)	Small molecule (15606394)	NSCLC	A kinase inhibitor of the EGFR family, including HER1, HER2, HER3, and HER4	Oral
Alectinib (Alecensa)	Small molecule (49806720)	NSCLC	Inhibits EML4-ALK fusion protein	Oral
Amivantamab-vmjw (Rybrevant)	Monoclonal antibody	NSCLC	Binds to EGFR and MET, preventing their ligands from binding	Intravenous
Atezolizumab (Tecentriq)	Monoclonal antibody	NSCLC, SCLC, melanoma, ASPS, HCC	Binding to PD-L1 prevents its interaction with PD-1 and B7-1 (CD80), promoting anti-tumor responses by immune cells	Intravenous
Binimetinib (Mektovi)	Small molecule (10288191)	NSCLC, melanoma	A kinase inhibitor of MEK1/2	Oral
Brigatinib (Alunbrig)	Small molecule (68165256)	NSCLC	A kinase inhibitor of multiple RTKs, including ALK, ROS1, IGF1R, EGFR, and FLT3	Oral
Capmatinib hydrochloride (Tabrecta)	Small molecule (122201352)	NSCLC	Inhibits MET kinase	Oral
Cemiplimab-rwlc (Libtayo)	Monoclonal antibody	NSCLC, BCC, cSCC	Blocks the PD-1 receptor on T cells, resulting in stimulation of the immune system	Intravenous
Ceritinib (Zykadia)	Small molecule (57379345)	NSCLC	Inhibits EML4-ALK fusion protein and ROS1 kinase	Oral
Crizotinib (Xalkori)	Small molecule (11626560)	NSCLC, ALCL, IMT	Inhibits EML4-ALK fusion protein, MET and ROS1 kinases	Oral
Dabrafenib mesylate (Tafinlar)	Small molecule (44516822)	NSCLC, melanoma, low grade glioma, solid tumors, ATC	Inhibits B-Raf with V600E, V600K, and V600D mutations	Oral
Dacomitinib (Vizimpro)	Small molecule (11511120)	NSCLC	A kinase inhibitor of the EGFR family, including HER1, HER2, and HER4	Oral
Docetaxel (Taxotere)	Small molecule (148124)	NSCLC, breast cancer, prostate cancer, HNSCC, GEJ	Binds to the β-subunit of tubulin, stabilizing microtubules and preventing their disassembly, thereby interfering with cell division	Intravenous
Durvalumab (Imfinzi)	Monoclonal antibody	NSCLC, SCLC, BTC, HCC, endometrial cancer	Binding to PD-L1 prevents its interaction with PD-1 and B7-1 (CD80), promoting anti-tumor responses by immune cells	Intravenous
Encorafenib (Braftovi)	Small molecule (50922675)	NSCLC, melanoma, CRC	Inhibits B-Raf^V600E^	Oral
Entrectinib (Rozlytrek)	Small molecule (25141092)	NSCLC, solid tumors	A kinase inhibitor of RTKs including Trks, ROS1, ALK	Oral
Gefitinib (Iressa)	Small molecule (123631)	NSCLC	Inhibits EGFR kinase	Oral
Ipilimumab (Yervoy)	Monoclonal antibody	NSCLC, CRC, melanoma, HCC, RCC, esophageal cancer, MPM	Binding to the CTLA-4 receptor on T cells prevents its interaction with B7-1 (CD80) and B7-2 (CD86), preventing inhibition of T cell-mediated immune response	Intravenous
Lazertinib mesylate hydrate (Lazcluze)	Small molecule (163203547)	NSCLC	Inhibits EGFR kinase	Oral
Lorlatinib (Lorbrena)	Small molecule (71731823)	NSCLC	Inhibits EML4-ALK fusion protein	Oral
Methotrexate sodium (Trexall, Xatmep)	Small molecule (11329481)	NSCLC, breast cancer, ALL, GTD, HNC, lung cancer, MF, NHL, osteosarcoma	Inhibits enzymes responsible for nucleotide synthesis including TS, DHFR, AICARFT, APRT	Oral or Intravenous
Necitumumab (Portrazza)	Monoclonal antibody	Squamous NSCLC	Binds to EGFR, preventing its ligand from binding	Intravenous
Nivolumab (Opdivo)	Monoclonal antibody	NSCLC, melanoma, CRC, cHL, GEJ, MPM, RCC, HNSCC, urothelial cancer	Binding to the PD-1 receptor on T cells prevents its interaction with PD-L1 and PD-L2, promoting anti-tumor immune responses	Intravenous
Osimertinib mesylate (Tagrisso)	Small molecule (78357807)	NSCLC	Inhibits EGFR kinase	Oral
Pembrolizumab (Keytruda)	Monoclonal antibody	NSCLC, breast cancer, melanoma, solid tumors, endometrial cancer, cervical cancer, BTC, cSCC, cHL, HCC, GEJ, RCC, HNSCC, urothelial cancer, MPM, MCC, PMBCL, MSI-H/dMMR	Binding to the PD-1 receptor on T cells prevents its interaction with PD-L1 and PD-L2, promoting anti-tumor immune responses	Intravenous
Pemetrexed disodium (Alimta, Pemfexy)	Small molecule (135413520)	Non-squamous NSCLC, MPM	Targets enzymes including TS, DHFR, GARFT, and AICARFT, resulting in disruption of DNA and RNA synthesis and protein production	Intravenous
Pralsetinib (Gavreto)	Small molecule (129073603)	NSCLC, thyroid cancer	Inhibits RET kinase	Oral
Ramucirumab (Cyramza)	Monoclonal antibody	NSCLC, CRC, HCC, GEJ	Binds to VEGFR2, preventing VEGF ligands (VEGF-A, VEGF-C, and VEGF-D) from binding	Intravenous
Repotrectinib (Augtyro)	Small molecule (135565923)	NSCLC, solid tumors	Inhibits ROS1 and Trks kinases	Oral
Selpercatinib (Retevmo)	Small molecule (134436906)	NSCLC, MTC, solid tumors, thyroid cancer	Inhibits RET kinase	Oral
Tepotinib hydrochloride (Tepmetko)	Small molecule (46700774)	NSCLC	Inhibits MET kinase	Oral
Sotorasib (Lumakras)	Small molecule (137278711)	NSCLC	A covalent inhibitor of K-Ras^G12C^	Oral
Trametinib dimethyl sulfoxide (Mekinist)	Small molecule (50992434)	NSCLC, melanoma, low grade glioma, solid tumors, ATC	An allosteric inhibitor of MEK1/2	Oral
Trastuzumab deruxtecan (Enhertu)	Monoclonal antibody	NSCLC, breast cancer, GEJ, solid tumors	As an ADC, binds to HER2 on the surface of tumor cells and upon internalization releases a drug (deruxtecan) that targets Topo I and induces cell death	Intravenous
Tremelimumab-actl (Imjudo)	Monoclonal antibody	NSCLC, HCC	Binding to the CTLA-4 receptor on T cells prevents its interaction with B7-1 (CD80) and B7-2 (CD86), promoting T cell-mediated cytotoxicity	Intravenous
Vinorelbine tartrate (Navelbine)	Small molecule (11607738)	NSCLC	Binds to tubulin, inhibits microtubule assembly and mitosis at metaphase	Intravenous

Drugs commonly used in other cancers listed in the tables above are not included. These NSCLC drugs are: doxorubicin hydrochloride (see Table 1); bevacizumab (see Table 2); erlotinib hydrochloride, everolimus, gemcitabine hydrochloride, paclitaxel albumin (see Table 3)

*ACK* activated Cdc42-associated kinase, *ADC* antibody-drug conjugate, *AICARFT* aminoimidazole carboxamide ribonucleotide formyltransferase, *ALCL* anaplastic large cell lymphoma, *APRT* amido phosphoribosyltransferase, *ASPS* alveolar soft part sarcoma, *ATC* anaplastic thyroid cancer, *BCC* basal cell carcinoma, *BTC* biliary tract cancer, *cHL* classic Hodgkin lymphoma, *cSCC* cutaneous squamous cell carcinoma, *DHFR* dihydrofolate reductase, *EML4* echinoderm microtubule-associated protein-like 4, *FAK* focal-adhesion kinase, *FLT3* fms-like tyrosine kinase 3, *GARFT* glycinamide ribonucleotide formyltransferase, *GEJ* gastroesophageal junction adenocarcinoma, *GTD* gestational trophoblastic disease, *HCC* hepatocellular carcinoma, *HNC* head and neck cancer, *HNSCC* squamous cell carcinoma of the head and neck, *IGF1R* insulin-like growth factor 1 receptor, *IMT* inflammatory myofibroblastic tumor, *LTK* leukocyte receptor tyrosine kinase, *MCC* merkel cell carcinoma, *MF* mycosis fungoides, *MPM* malignant pleural mesothelioma, *MSI-H/dMMR* microsatellite instability-high (MSI-H) or mismatch repair deficient (dMMR) cancer, *MTC* medullary thyroid cancer, *NHL* non-Hodgkin lymphoma, *PD-L1* programmed cell death-ligand 1, *PMBCL* primary mediastinal large B-cell lymphoma, *RCC* renal cell carcinoma, *Topo I* topoisomerase I, *TS* thymidylate synthase, *VEGFR2* vascular endothelial growth factor receptor 2

The FDA has approved a significant number of drugs for the treatment of leukemia, including ALL, AML, CLL and CML.^393^ Furthermore, a number of drugs have been approved for use in the treatment of AML only (Table 5). The FDA also approved a combination form of daunorubicin hydrochloride and cytarabine contained inside liposome (Vyxeos) for AML. Idarubicin is a structural analog of daunorubicin with the same mechanism of action. Drug combinations used in AML include ADE (cytarabine + daunorubicin hydrochloride + etoposide phosphate).^393^

**Table 5:** FDA-approved drugs for AML

Drug name (brand name)	Drug type (PubChem CID)	Cancer treatment	Mechanism of action	Route of administration
Arsenic trioxide (Trisenox)	Small molecule (14888)	APL	Induces apoptosis, promotes cell differentiation, suppresses cell proliferation	Intravenous
Azacitidine (Onureg, Vidaza)	Small molecule (9444)	AML, myelodysplastic syndromes	Inhibits DNA methyltransferase, resulting in hypomethylation of DNA, and incorporates into RNA, resulting in disassembly of polyribosomes, defective methylation and acceptor function of transfer RNA, and inhibition of protein production	Intravenous or subcutaneous
Cytarabine (Cytosar)	Small molecule (6253)	AML, meningeal leukemia, ALL, CML	Inhibits nucleic acid synthesis and DNA polymerase, blocking the cell cycle progression from G1 to S phase	Intravenous, subcutaneous or intrathecal
Daunorubicin hydrochloride (Cerubidine)	Small molecule (62770)	AML, ALL	Intercalates between the DNA base pairs, causing the double-strand unwinding via inhibition of Topo II, resulting in single- and double-strand breaks, thus inhibiting DNA and RNA synthesis	Intravenous
Dexamethasone (Alba-Dex, Baycadron, Decadrol, Decadron, Dextenza, Decasone, Deronil, Dexameth, Dxevo, Gammacorten, Hemady, Hexadrol, Ozurdex, TaperDex, ZoDex)	Small molecule (5743)	Leukemia, lymphoma, multiple myeloma, mycosis fungoides	As a glucocorticoid agonist, binds to GR and translocates to the nucleus to bind GREs, leading to suppress inflammatory cytokines and inducing apoptosis	Oral, intravenous or intramuscular
Enasidenib mesylate (Idhifa)	Small molecule (90480031)	AML	Selectively inhibits IDH2 mutants, resulting in reduced production of the oncometabolite D2HG, restoration of cell differentiation and clonal proliferation of myeloid lineage cells	Oral
Gemtuzumab ozogamicin (Mylotarg)	Monoclonal antibody	AML	As an ADC, binds to CD33 on the surface of leukemia cells and releases a drug (calicheamicin derivative) upon internalization, resulting in site-specific DNA double-strand breaks and cell death	Intravenous
Gilteritinib fumarate (Xospata)	Small molecule (76970819)	AML	Inhibits the internal tandem tyrosine kinase domain of FLT3 and also inhibits AXL and ALK	Oral
Glasdegib maleate (Daurismo)	Small molecule (122640033)	AML	Binds to the SMO receptor, inhibiting the Hedgehog signaling pathway	Oral
Idarubicin hydrochloride (Idamycin)	Small molecule (636362)	AML	Intercalates between the DNA base pairs, causing the double-strand unwinding via inhibition of Topo II, resulting in single- and double-strand breaks, thus inhibiting DNA and RNA synthesis	Oral or intravenous
Ivosidenib (Tibsovo)	Small molecule (71657455)	AML, CCA, myelodysplastic syndrome	Inhibits IDH1 mutants, resulting in reduced production of the oncometabolite D2HG and promotion of cell differentiation	Oral
Midostaurin (Rydapt)	Small molecule (9829523)	AML, ASM, MCL, SM-AHN	Inhibits PKCα, VEGFR2, c-Kit, PDGFR, FLT3 tyrosine kinases	Oral
Mitoxantrone hydrochloride (Novantrone)	Small molecule (51082)	AML, prostate cancer	Intercalates between DNA base pairs, causing double-strand unwinding via inhibition of Topo II, resulting in disruption of DNA synthesis and repair	Intravenous
Olutasidenib (Rezlidhia)	Small molecule (118955396)	AML	Selectively inhibits IDH1 mutants, resulting in reduced production of the oncometabolite D2HG and promotion of cell differentiation	Oral
Pemigatinib (Pemazyre)	Small molecule (86705695)	AML, CCA, myeloid or lymphoid cancer	Inhibits FGFR1, FGFR2 and FGFR3 kinase	Intravenous
Prednisone (Deltasone)	Small molecule (5865)	AML, ALL, CLL, CML, HL, MF, NHL	As a glucocorticoid agonist, binds to GR with less affinity than dexamethasone and translocates to the nucleus to bind GREs, resulting in a milder anti-inflammatory effect	Oral
Quizartinib dihydrochloride (Vanflyta)	Small molecule (25184035)	AML	Inhibits FLT3 kinase	Oral
Thioguanine (Tabloid, Lanvis)	Small molecule (2723601)	AML	Utilizes HGPRTase to convert to TGMP, which is further converted by cellular kinases to active TGTP, which incorporates into DNA, leading to DNA strand breaks, cell cycle arrest and apoptosis	Oral
Venetoclax (Venclexta)	Small molecule (49846579)	AML, CLL, SLL	Selectively inhibits the anti-apoptotic BCL-2 on the surface of mitochondria, leading to apoptosis of cancer cells	Oral

*ADC* antibody-drug conjugate, *ASM* aggressive systemic mastocytosis, *BCL-2* B-cell lymphoma-2, *CCA* cholangiocarcinoma, *D2HG* D-2-hydroxyglutarate, *FLT3* fms-like tyrosine kinase 3, *GR* glucocorticoid receptor, *GRE* glucocorticoid response element, *HGPRTase* hypoxanthine-guanine phosphoribosyltransferase, *HL* Hodgkin lymphoma, *MF* mycosis fungoides, *MCL* mast cell leukemia, *NHL* non-Hodgkin lymphoma, *SLL* small lymphocytic lymphoma, *SMO* smoothened, *SM-AHN* systemic mastocytosis with associated hematologic neoplasm, *TGMP* thioguanine monophosphate, *TGTP* thioguanine triphosphate, *Topo II* topoisomerase II, *VEGFR2* vascular endothelial growth factor receptor 2

Drugs commonly used in other cancers listed in the tables above are not included. These AML drugs are: cyclophosphamide, doxorubicin hydrochloride, vincristine sulfate (see Table 1)

In the case of breast cancer, scores of drugs have been granted approval,^394^ with the list of these drugs too extensive to enumerate. Some of the drugs approved to treat breast cancer are also used to treat other types of cancer, including cyclophosphamide and doxorubicin hydrochloride for neuroblastoma; 5-fluorouracil, capecitabine, everolimus, gemcitabine hydrochloride, olaparib and paclitaxel albumin for pancreatic cancer; docetaxel, doxorubicin hydrochloride, everolimus, gemcitabine hydrochloride, methotrexate sodium, paclitaxel albumin, pembrolizumab and trastuzumab for NSCLC. The individual drugs in the combinations are FDA-approved. However, the drug combinations are commonly not approved, although widely used. The most recent FDA approved drug is ribociclib succinate (Kisqali) with an aromatase inhibitor for the adjuvant treatment of adults with hormone receptor (HR)-positive, HER2-negative stage II and III early breast cancer. Drug combinations used in breast cancer includes AC (doxorubicin hydrochloride + cyclophosphamide), AC-T (doxorubicin hydrochloride + cyclophosphamide + paclitaxel), CAF (cyclophosphamide + doxorubicin hydrochloride + fluorouracil), CMF (cyclophosphamide + methotrexate + fluorouracil), FEC (fluorouracil + epirubicin hydrochloride + cyclophosphamide), and TAC (docetaxel + doxorubicin hydrochloride + cyclophosphamide).^394^

Liver cancer is not included in our detailed discussion of aggressive cancers above, but it can be highly aggressive. Liver cancer is often unamenable to surgery since by the time it is diagnosed it has already spread or is intertwined with blood vessels. For liver cancer, the FDA approved atezolizumab (Tecentriq) and bevacizumab (Avastin).^395^ As expected, clinical studies indicated that their combination is better than the single therapies. Atezolizumab is an immune checkpoint inhibitor. Bevacizumab prevents growth of new blood vessels. The updated list of drugs approved for liver cancer includes the atezolizumab and bevacizumab discussed above.^396^ Drugs approved to treat HCC, a type of liver cancer, are also used to treat other types of cancer, including bevacizumab for glioblastoma; atezolizumab, bevacizumab, durvalumab, ipilimumab, pembrolizumab, ramucirumab and tremelimumab-actl for NSCLC. The drugs ivosidenib and pemigatinib, which are approved to treat AML, are also approved to treat cholangiocarcinoma (CCA), a rare and aggressive cancer that forms in the bile ducts. Developing from cells in the liver, liver cancer can be especially aggressive. There are a few types determined by the liver cell where it originated.^397^ The major types include (1) HCC, which is the most common. Eighty % of the liver cells are hepatocytes. Severe liver damage, or cirrhosis is believed to be the origin for this cancer. (2) Cholangiocarcinoma is a bile duct cancer. (3) Fibrolamellar carcinoma is very rare, occurring in people with healthy livers. (4) Hepatoblastoma is an extremely rare type of liver cancer but common in young children. (5) Liver angiosarcoma also rare, forms in blood cells or lymph vessels, part of your immune system.

CRC develops slowly, and if caught early, is treatable. However, it is the second deadliest cancer in the U. S., with its metastasis posing a challenge, primarily due to limited therapeutic options. Optimal treatment depends on the tumor type. High-grade large cell and small cell neuroendocrine tumors are aggressive. Metastatic CRC (mCRC) tends to be relatively more lethal than primary CRC. 30% of colorectal cancers are mesenchymal mCRC, with hyaluronan accretion a key step in mCRC tumors formation, especially under low levels of PKCζ and PKCι, resulting in worse prognoses.^398^ A combination of anti-PD-L1 and anti-CTLA-4 antibodies with hyaluronidase, which breaks down hyaluronan, has been investigated as a potential treatment for targeting CRCs that metastasized to the liver, which is a common occurrence, making hyaluronidase an important agent for immunotherapy success. A recent review^398^ discussed the genetic alterations associated with advanced CRC and metastasis as well as the role of cellular heterogeneity and plasticity in drug resistance and metastasis and how the tumor microenvironment impacts it. However, beyond generic concepts, much is still not understood, hampering effective pharmacologic treatment. The FDA has approved a number of drugs for the treatment of colon and rectal cancer.^399^ Broadly, mesenchymal stromal cells (MSCs) are constituents of tumor stroma.^400^ They promote a favorable environment for tumor progression, proliferation, and metastasis, clarifying why metastatic mCRC is often lethal.

Above, we overviewed currently available and prescribed single drugs, and drug combinations. In the interest of brevity, we did not detail the ages of the patients, prior treatments, etc., which are provided at the links that we cited.

### Clinical research progress: treatment design

Here we take up the challenging question of directions for future research and treatment designs for highly aggressive cancers. We consider the rational, available relevant tools and data, and expanding their applications. The question is which combination of targets to consider, which can guide drug combinations. With this goal in mind, we suggest considering that primary cancers are tissue specific, and that highly aggressive cancers present an extensive heterogeneity, proliferation, and metastases. This argues for harnessing spatial single-cell transcriptomic to identify microclones, which are subpopulations of cancer cells that exist within tumors, and analysis of the cancer-specific metastases to identify the tissues that the cancer favors for its metastases to settle in.^401–403^ Data inform that specific cancers prefer certain host tissues. This leads us to suggest that besides comprehensive searches to identify the metastases, analyses of the overexpressed genes be carried out, as these can provide clues as well. Early data by Axelsen et al.^404^ observed that at least some cancers tend to metastasize in tissues which express those overexpressed genes. They suggested, and we believe, that this is likely to be a general trend. These data can couple with statistical trends of cancer-specific co-occurring driver mutations, mapped on their harboring proteins and pathways. Drug resistance commonly exploits parallel pathways and bypasses engaged through pathway crosslinkages, which are overflooded in aggressive cancers. Spatial single-cell transcriptomic is crucial to diagnose overexpression, which commonly involves epigenetic alteration, not identifiable via mutational analyses.

In addition to identifying pathways by mapping of mutations, pathway-based approaches are currently being used to study cancer in biological networks, including Gene Set Enrichment (GSE), which identifies pathways enriched for genes with altered expression;^405^ Over-Representation Analysis (ORA), which identifies pathways with more differentially expressed genes than would be expected by chance; Function Class Scoring (FCS), which identifies disease pathways based on the aggregate of their gene expression values; Differential Causal Effects (DCE), which identifies dysregulated signaling pathways in cancer cells and compares normal and cancer cells using the statistical framework of causality;^406^ and CTpathway, which identifies cancer target pathways in early-stage tissues and blood samples.^407^

Collectively, our treatment designs against aggressive cancers innovates by offering research involving analyses of cancer-specific metastases, and primary and metastases-specific tumor single-cell spatial data, and further supporting broad pathway analyses along with transcription factors and epigenetic modulators. Our on-going work harnesses an innovative pathway-mapping strategy.

## Conclusions

Here we asked: *What are the underlying molecular principles of aggressive cancers*? *What distinguishes them from the less aggressive, more treatable ones*? And crucially, *can their molecular hallmarks offer clues to anti-cancer drug combination strategies targeting their ultra-strong drug resistance*?

Our review took up these questions, noting that whereas several factors can be in play,^1–5^ the accelerated growth and malignancy of aggressive cancers point to a massive payload of primed, catalysis-ready states resulting from unmitigated overexpression of oncogenic proteins, and high load of activation mutations. High population of these oncogenic signaling nodes activates not only its functionally intended pathways, but multiple oncogenic pathways, as shown in the case of *PIK3CA*,^18^ fostering heterogeneity and proliferation. In glioblastoma, the mutations alter EGFR dimer formation, attenuating ligand bias, corrupting downstream signaling.^408^ Mutations that alter the transcriptional landscapes, perturb the protein interactome,^409,410^ increase cancer heterogeneity and aggressiveness. High oncogenic mutational loads, and overexpression, break homeostatic physiological feedback mechanisms.^322,411^

The National Cancer Institute defined aggressive cancer as one that spreads rapidly, despite severe treatment. *Here we offer the underlying principle on the fundamental structural level*: *Aggressive cancers are those harboring an exceedingly large population of proteins critical in signal transduction in major proliferation pathways in their active, catalysis-prone states*. That is, the structures of these dominant signaling proteins are in their active conformations, primed to execute their signaling roles.^412^ This definition implicitly articulates overexpression of master transcription factors, transcription factors with gene fusions, copy number alterations, and dysregulation of the epigenetic codes, especially in super-enhancers. High mutation loads of vital upstream signaling regulators (Fig. 1), such as RTKs and K-Ras, also fits into this definition. *A combination* of overexpression of multiple such proteins, epigenetic alterations, and activating mutations of major upstream signaling nodes can be decisive, suggesting that acquisition of these attributes can serve as hallmark of aggressive cancers. Their impact can be quantified by signaling by-the-numbers scenarios, where the numbers refer to the number of active state conformations.^13^

While the same protein may populate many cancers, its abundance and the predominant mutation may vary, depending on the cell type, state, cell history, the background and evolving mutational load, with epigenetics and chromatin accessibility of the respective genes playing major roles.^43,413^ Heterogeneity implies that resistance mutations are likely to exist prior to treatment. As discussed here, drugs harnessed to target aggressive cancers may overlap, but also be distinct aiming at the specific proteins and their more frequent mutations.

Forecasting these can assist the attending oncologist.^24,414,415^ As to *anti-cancer drug combination strategies*, mapping the drugs onto the respective signaling pathways, may indicate their type of complementarity, and how it might be improved.^57,58^ Coupling it with pathway mapping of the oncogenic proteins whose expression indicates substantial deviation from that of normal tissues, and those that are highly mutated, may better inform combinatorial selection. These should be coupled with drugs targeting epigenetics regulators, master transcription factors, fused transcription factors, and those with copy number variations.

To conclude, here we propose that the absolute number of active (oncogenic) conformations that the cancer harbors are a foundational hallmark of its aggressiveness (Fig. 1). The higher the number—the more overspilled the signaling—the higher the heterogeneity. In aggressive cancers the number is extremely high. We call this hallmark “cancer aggressiveness by-the-numbers”. We designate it “foundational” since it is expressed on the molecular level by conformational distributions, the most fundamental physical-chemical attribute of biomacromolecules. Dynamic conformational, or ensemble, propensities decide molecular, and cell function.^37,38^
